# Diagnostic accuracy of the Xpert MTB/RIF assay for bone and joint tuberculosis: A meta-analysis

**DOI:** 10.1371/journal.pone.0221427

**Published:** 2019-08-22

**Authors:** Yanqin Shen, Guocan Yu, Fangming Zhong, Xiaohua Kong

**Affiliations:** Zhejiang Tuberculosis Diagnosis and Treatment Center, Zhejiang Chinese Medicine and Western Medicine Integrated Hospital, Hangzhou, Zhejiang, China; University of Mississippi Medical Center, UNITED STATES

## Abstract

**Background:**

This study aimed to evaluate the accuracy of the Xpert MTB/RIF assay for the diagnosis of bone and joint tuberculosis.

**Methods:**

We searched databases from their inception to May 7, 2019 for published articles and reviewed them to assess the accuracy of Xpert MTB/RIF with respect to a composite reference standard (CRS) and mycobacterial culture. Meta-analyses were performed using a bivariate random-effects model, and the sources of heterogeneity were assessed via subgroup analysis and meta-regression.

**Results:**

Nineteen independent (9 prospective, 5 retrospective, and 5 case-control) studies that compared Xpert MTB/RIF with the CRS and 14 (6 prospective, 7 retrospective, and 1 case-control) studies that compared it with culture were included. The pooled sensitivity and specificity of Xpert MTB/RIF were 81% (95% confidence interval [CI], 77–84) and 99% (95% CI, 97–100) compared to the CRS, respectively, and 96% (95% CI, 90–98) and 85% (95% CI, 57–96) compared to culture, respectively. The pooled sensitivity and specificity using pus samples vs. the CRS were 82% (95% CI, 76–86) and 99% (95% CI, 95–100), respectively. The proportions obtained while working with tissue samples vs. the CRS were 84% (95% CI, 76–90) and 98% (95% CI, 94–99), respectively. There was no significant difference in diagnostic accuracy among the types of specimens.

**Conclusions:**

Xpert MTB/RIF demonstrates good diagnostic accuracy for bone and joint tuberculosis, the results of which are not related to the type of specimen.

## Introduction

Tuberculosis is a major infectious disease globally and poses a serious threat to public health [1]. Extrapulmonary tuberculosis (EPTB) accounts for about 10% of all tuberculosis cases [1]. Bone and joint tuberculosis (BJTB) is a common type of EPTB and accounts for about 10–34% of EPTB cases [2, 3]. BJTB can lead to joint destruction, deformity, and even paraplegia, thereby seriously affecting the quality of life. Therefore, early and correct diagnosis and treatment are critical [4]. However, early diagnosis is very difficult due to the atypical symptoms of BJTB, deep lesions, and difficulty in obtaining specimens [5]. Traditional diagnostic protocols, such as *Mycobacterium tuberculosis* culture, are quite time-consuming and have a low sensitivity [6]. Therefore, rapid laboratory diagnosis of BJTB is an urgent necessity. The Xpert MTB/RIF assay is a rapid, automated molecular test with a high accuracy for the detection of pulmonary tuberculosis (PTB) and EPTB [7]. This assay has also been recommended for the diagnosis of lymph node tuberculosis and has shown good diagnostic accuracy [8]. However, the diagnostic accuracy of this assay for BJTB remains controversial. Due to the lack of independent systematic research on the diagnostic accuracy of Xpert MTB/RIF assay for BJTB, the possible influence of the type of specimen (pus and tissue samples) on the results is yet to be clarified. Therefore, we performed a meta-analysis to confirm the diagnostic accuracy of the Xpert MTB/RIF assay, compared to that of the composite reference standard (CRS) and mycobacterial culture, in the detection of BJTB. We assessed the pooled sensitivity and specificity of this assay compared to different references. Moreover, the diagnostic accuracy of the test was evaluated, based on different sample types, lesion sites, conditions of samples, and patient selection methods by subgroup analysis.

## Materials and methods

### Data sources and search strategy

We searched PubMed, Embase, the Cochrane Library, the Wanfang database, and China National Knowledge Infrastructure for studies evaluating the diagnostic accuracy of Xpert for BJTB on May 7, 2019. The search formula ((Xpert OR Gene Xpert) AND ("Tuberculosis, Osteoarticular"[Mesh] OR "Tuberculosis, Spinal"[Mesh] OR "Extra pulmonary tuberculosis")) was used for PubMed without any limitation. Similar search formulae were used for Embase, the Cochrane Library, China National Knowledge Infrastructure, and Wanfang databases. References cited in the included articles and reviews were further explored for possible candidate studies.

### Inclusion criteria

We included full-text original studies that assessed the diagnostic accuracy of Xpert assay for BJTB using bone and joint specimens. Reference standards were well-defined and appropriate to the studies. The articles directly provided true positive (TP), false positive (FP), false negative (FN), and true negative (TN) values for the assay or included the data necessary to calculate these measures. We excluded case reports, articles written in languages other than English and Chinese, studies with < 10 samples, conference reports, and abstracts without full articles.

### Reference standard

A composite reference standard (CRS) or mycobacterial culture was defined as the reference standard in our study. Clinical symptoms, radiographic features, biochemical test results, smears, culture, histopathology, and response to anti-tuberculosis drugs constituted the reference standards in the CRS. Some or all of the factors with positive results were considered positive for BJTB. Cases were considered as non-BJTB if all the results were negative. We used the CRS as defined in the original paper.

### Literature screening and selection

Two investigators (Guocan Yu and Yanqin Shen) independently assessed the candidate articles by reviewing their titles and abstracts, followed by the full text, for inclusion. Discrepancies between the two investigators were resolved by discussion with a third investigator (Xiaohua Kong).

### Data extraction

We extracted data including author name; year; country; TP, FP, FN, and TN values for the assay; reference standard; patient selection method; some steps (e.g., homogenization); specimen type; and condition along with other parameters. The same two investigators independently extracted the necessary information from each of the included articles; we cross-checked the information they obtained. Discrepancies in the two data sets were settled by a discussion with a third investigator, similar to that used during the literature selection phase. Data from studies against two different reference standards were treated separately.

### Assessment of study quality

Based on the two reference standards (CRS and culture), the two investigators independently divided the studies into two groups and used a revised tool for Quality Assessment of Diagnostic Accuracy Studies (QUADAS-2) to assess study quality separately [9]. We chose not to carry out formal assessment of publication bias, as the available methods such as funnel plots are not considered valid for diagnostic accuracy reviews [10].

### Data synthesis and statistical analysis

We first obtained the values corresponding to TP, FP, FN, and TN in each included study, and calculated the estimated pooled sensitivity and specificity of Xpert MTB/RIF associated with the 95% confidence interval (CI), against CRS or culture, using bivariate random-effects models. Forest plots for sensitivity and specificity were generated for each study. The areas under summary receiver operating characteristic (SROC) curves (AUC) were subsequently calculated. *I*^2^ statistics were used to assess heterogeneity between the studies and a reference standard. While 0% indicated no observed heterogeneity, values greater than 50% were considered to imply substantial heterogeneity [11]. We explored different types of samples, different lesion sites, different patient selection method, decontamination methods, sample conditions, and homogenization as potential sources of heterogeneity, using subgroup and meta-regression analyses. At least four published studies were required to perform the meta-analysis for predefined variable types. Data from studies against CRS and culture were analyzed separately. Stata version 14.0 (Stata Corp., College Station, TX, USA) with the *midas* command packages was used to generate forest plots of sensitivity and specificity with 95% CI for each study and carry out meta-analyses and meta-regression analyses.

### Imperfect reference standards

Imperfect reference standards may lead to misclassification of samples in diagnostic validity studies [12, 13]. Due to the paucibacillary nature of EPTB, a culture would be an imperfect reference standard and lead to an underestimation of the true specificity of Xpert MTB/RIF. A CRS is a composite standard that comprises results from several tests; however, a CRS itself may have reduced specificity, thereby leading to apparent FN Xpert MTB/RIF results, an underestimation of the true sensitivity of Xpert MTB/RIF [12, 14]. Therefore, a study comparing Xpert MTB/RIF with both culture and CRS might provide a more credible range for sensitivity and specificity.

## Results

### Identification of studies and study characteristics

**Fig 1:**
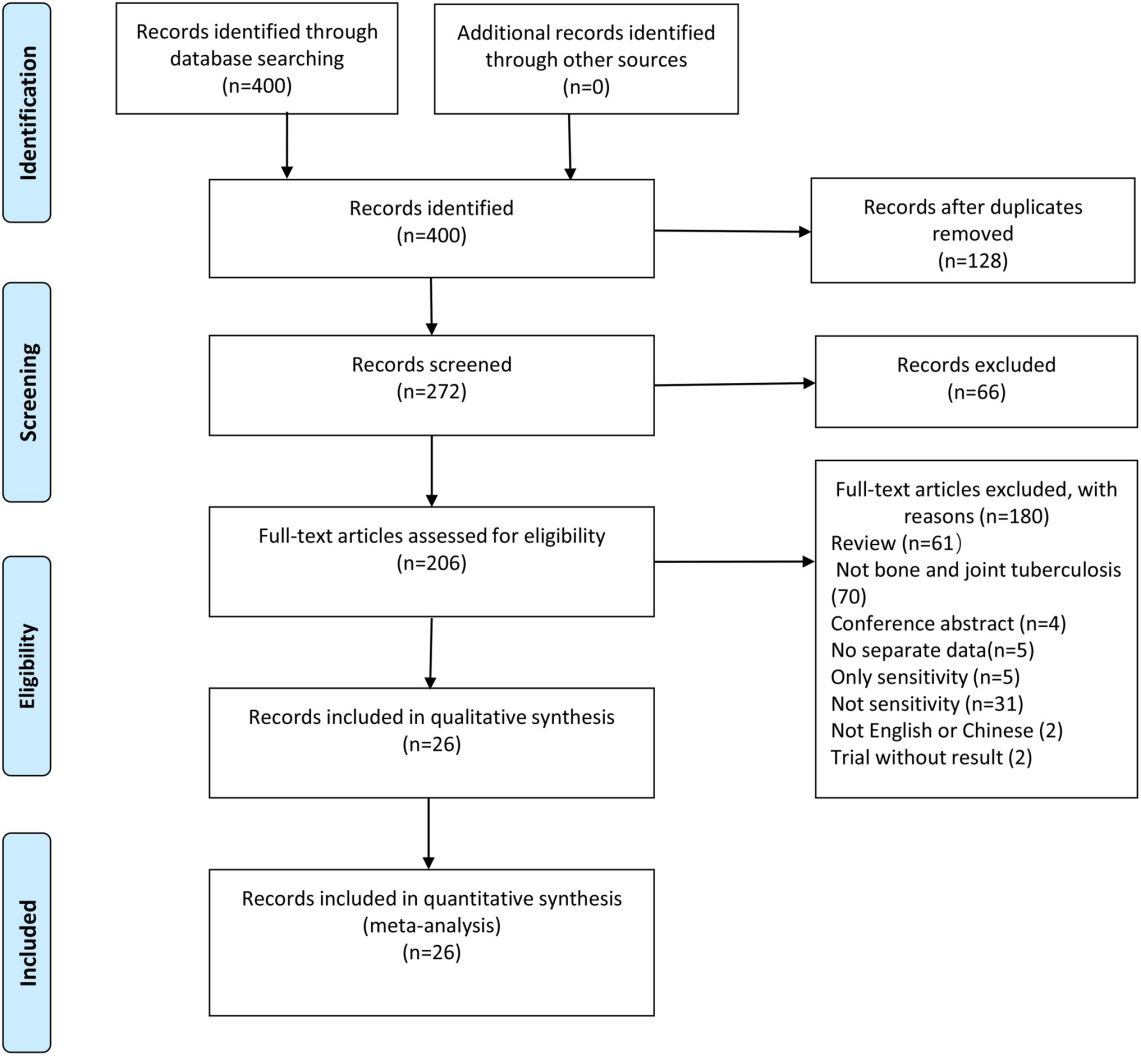
Literature retrieval flow chart. 124, 22, 228, 15, 11 articles were found from Pubmed, the Cochrane Library, Embase, Wanfang database and CNKI respectively.

Through our search strategy, four hundred candidate articles were identified from relevant databases, and twenty-six qualified articles were included according to the inclusion criteria (Fig 1) [15–40], including 12 prospective studies, 9 retrospective studies and 5 case-control studies. The kappa index of agreement for the selection and data extraction was 0.877 (95% CI, 0.779–0.975) between the two investigators. Only two studies were conducted in high-income countries [18, 35], while the rest were conducted in low- and middle-income countries. Fifteen articles were written in English and eleven in Chinese. A median of 109 specimens was evaluated in each article (range, 13–418). We excluded five other articles that reported on the sensitivity only, without reporting any specificity [41–45]. Two other articles published in languages other than English or Chinese were excluded [46, 47]. The specimens used in the studies included pus, tissue, and joint fluid. The commonest site of infection was the spine.

Articles that reported the use of two different reference standards in the same study were considered to include two independent studies. In accordance with this principle, 33 independent studies were included: 19 (9 prospective studies, 5 retrospective studies, and 5 case-control studies) compared Xpert MTB/RIF with CRS, and 14 (6 prospective studies, 7 retrospective studies, and 1 case-control study) compared Xpert MTB/RIF with culture (Table 1).

**Table 1:** Characteristics of the included studies.

Author	Year	County	Sample type	Reference	TP	FP	FN	TN	Decontaminate method	Sample condition	Homogenisation	SR	Patient selection method
Held, M.a	2014	South Africa	spinal tissue	culture	25	9	2	35	No	fresh	Mechanical	3:1	consecutive
Held, M.b	2014	South Africa	spinal tissue	crs	44	1	2	25	No	fresh	Mechanical	3:1	consecutive
Litao Li	2014	China	spinal tissue or pus	culture	63	26	1	50	NALC-NaOH	frozen	Mechanical	-	Convenience
Gu,Y.a	2015	China	bone and joint pus	crs	41	0	9	10	No	fresh	No	2:1	Convenience
Gu,Y.b	2015	China	bone and joint pus	culture	24	17	0	19	No	fresh	No	2:1	Convenience
Kim, Y. W.a	2015	South Korea	joint fluid	culture	3	0	0	280	NALC-NaOH	frozen	No	-	consecutive
Kim, Y. W.b	2015	South Korea	joint fluid	crs	3	0	0	280	NALC-NaOH	frozen	No	-	consecutive
Hongmei Chen	2015	China	bone and joint pus	crs	100	0	30	9	No	fresh	No	-	Convenience
Wenyun Jia	2015	China	bone and joint pus	crs	46	1	3	31	No	fresh	No	2:1	Convenience
Held, M.	2016	South Africa	bone and joint tissue	crs	17	0	6	86	No	fresh	No	3:1	consecutive
Zhen Li.a	2016	China	bone and joint tissue or pus	crs	199	0	54	59	NALC-NaOH	fresh	Mechanical	2:1	Convenience
Zhen Li.b	2016	China	bone and joint tissue or pus	culture	121	78	1	112	NALC-NaOH	fresh	Mechanical	2:1	Convenience
Kai Tang	2016	China	knee joint fluid	crs	20	2	9	18	No	fresh	No	2:1	consecutive
Arockiaraj, J.a	2017	India	spinal tissue or pus	crs	180	0	73	85	NALC-NaOH	fresh	No	-	Convenience
Arockiaraj, J.b	2017	India	spinal tissue or pus	culture	99	82	13	144	NALC-NaOH	fresh	No	-	Convenience
Held, M.	2017	South Africa	bone and joint tissue	crs	84	1	7	114	No	fresh	No	3:1	consecutive
Jin, Y. H.	2017	China	spinal pus	crs	63	0	18	28	No	fresh	Mechanical	2:1	Convenience
Li, Y.	2017	China	joint fluid	culture	26	2	1	6	No	fresh	No	2:1	Convenience
Massi, M. N.	2017	Indonesia	vertebral bone tissues.	culture	22	40	0	8	NALC-NaOH	fresh	No	2:1	Convenience
Tang, L.	2017	China	spinal tissue or pus	culture	96	57	3	67	NALC-NaOH	frozen	No	3:1	Convenience
Weijie Dong	2017	China	bone and joint tissue	crs	196	0	55	21	NALC-NaOH	fresh	Mechanical	2:1	Convenience
Zheng Zhou	2017	China	spinal tissue	crs	55	1	17	19	No	fresh	Mechanical	2:1	Convenience
Zhujun Zhu	2017	China	bone and joint pus	crs	55	1	12	29	No	fresh	Mechanical	2:1	Convenience
Khan, A. S.a	2018	Pakistan	joint fluid	culture	6	0	1	6	No	fresh	No	-	Convenience
Khan, A. S.b	2018	Pakistan	Bone marrow	culture	3	0	3	30	No	fresh	No	-	Convenience
Li, Y.	2018	China	bone and joint pus	crs	24	0	12	13	NALC-NaOH	fresh	Mechanical	2:1	consecutive
Perez-Risco, D.	2018	Spain	bone and joint tissue or pus	culture	14	0	3	23	No	frozen	No	2:1	Convenience
Tang, Y.	2018	China	joint pus	crs	61	0	25	30	No	fresh	No		Convenience
Wang, G.	2018	China	spinal pus	crs	272	0	47	99	NALC-NaOH	fresh	No	2:1	Convenience
Tingting Hou	2018	China	spinal tissue	crs	155	4	37	46	NALC-NaOH	fresh	Mechanical	2:1.5	Convenience
Chenguang Jia	2018	China	spinal pus	culture	69	8	1	90	-	frozen	No	1.5:1	Convenience
Hongwei Liu.a	2018	China	bone and joint pus	crs	84	2	11	32	No	fresh	Mechanical	2:1	Convenience
Hongwei Liu.b	2018	China	bone and joint pus	culture	30	54	0	11	No	fresh	Mechanical	2:1	Convenience

CRS, composite reference standard; TP, true positive; FP, false positive; FN, false negative; TN, true negative; SR, sample ratio

### Study quality

**Fig 2:**
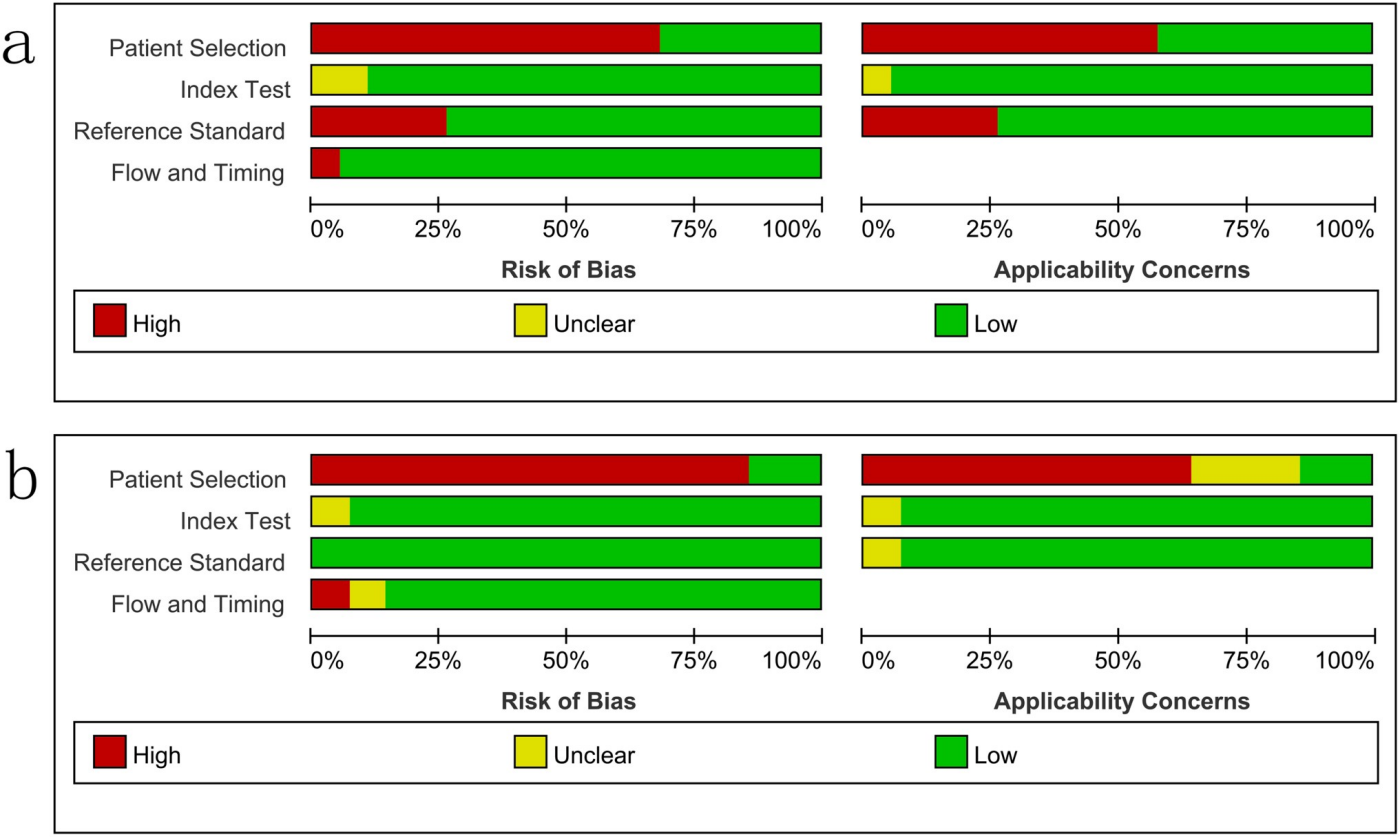
Methodological quality graphs (risk of bias and applicability concerns) as percentages across the included studies. a: composite reference standard. b: culture reference standard.

The overall methodological quality of the included studies, using a CRS and culture, is summarized in Fig 2. The risk of bias was mainly due to patient selection and the reference standard. The flow and timing of the risk of bias from the index test was judged to be relatively low.

### Diagnostic accuracy of Xpert MTB/RIF assay for BJTB

**Fig 3:**
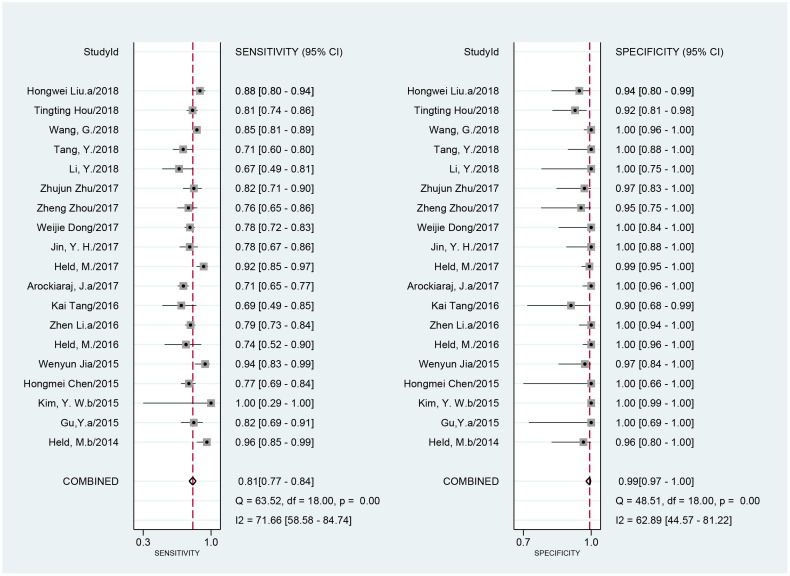
Forest plot of Xpert sensitivity and specificity for tuberculosis detection in BJTB compared with a composite reference standard. BJTB: bone and joint tuberculosis.

Nineteen studies included a comparison of 3173 samples with a CRS. The sensitivity of Xpert MTB/RIF ranged from 67% (95% CI, 49–81) to 100% (95% CI, 29–100). The pooled sensitivity of Xpert MTB/RIF assay for BJTB was 81% (*I*^2^ = 72%; 95% CI, 77–84) and the specificity ranged from 90% (95% CI, 68–99) to 100% (95% CI, 99–100). The pooled specificity of Xpert MTB/RIF was 99% (*I*^2^ = 63%; 95% CI, 97–100) (Fig 3). There was substantial heterogeneity of sensitivity and specificity. The AUC of the SROC was 0.93 (95% CI, 0.91–0.95).

**Fig 4:**
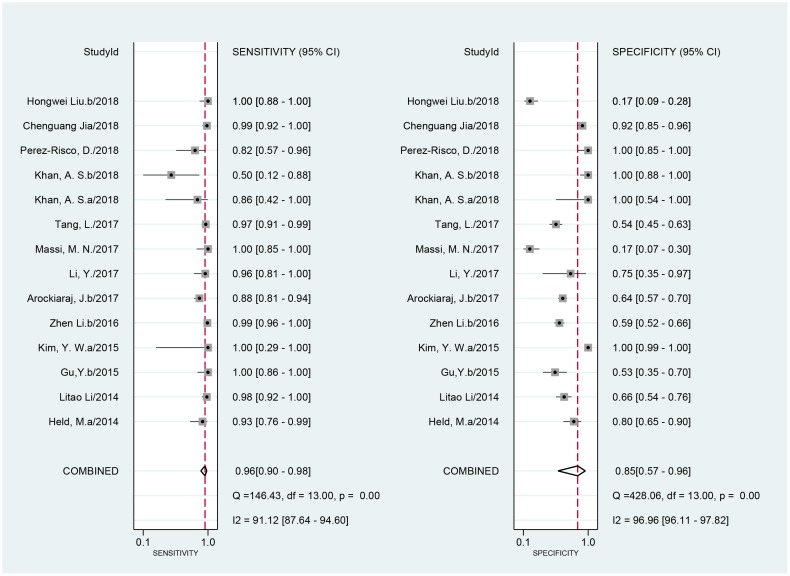
Forest plot of Xpert sensitivity and specificity for tuberculosis detection in BJTB compared with culture reference standard. BJTB: bone and joint tuberculosis.

When compared to a culture standard, 14 studies with 1884 samples were included. The sensitivity of Xpert MTB/RIF ranged from 50% (95% CI, 12–88) to 100% (95% CI, 88–100). The pooled sensitivity of Xpert MTB/RIF was 96% (*I*^2^ = 91%; 95% CI, 90–98). The specificity of Xpert MTB/RIF ranged from 17% (95% CI, 9–28) to 100% (95% CI, 99–100) and the pooled specificity was 85% (*I*^2^ = 97%; 95% CI, 57–96) (Fig 4). As expected, the sensitivity was improved, and the specificity was undervalued when compared to the culture. There was substantial heterogeneity of sensitivity and specificity. The AUC of the SROC was 0.97 (95% CI, 0.96–0.98) vs. that of the culture, suggesting an excellent overall diagnostic accuracy.

**Fig 5:**
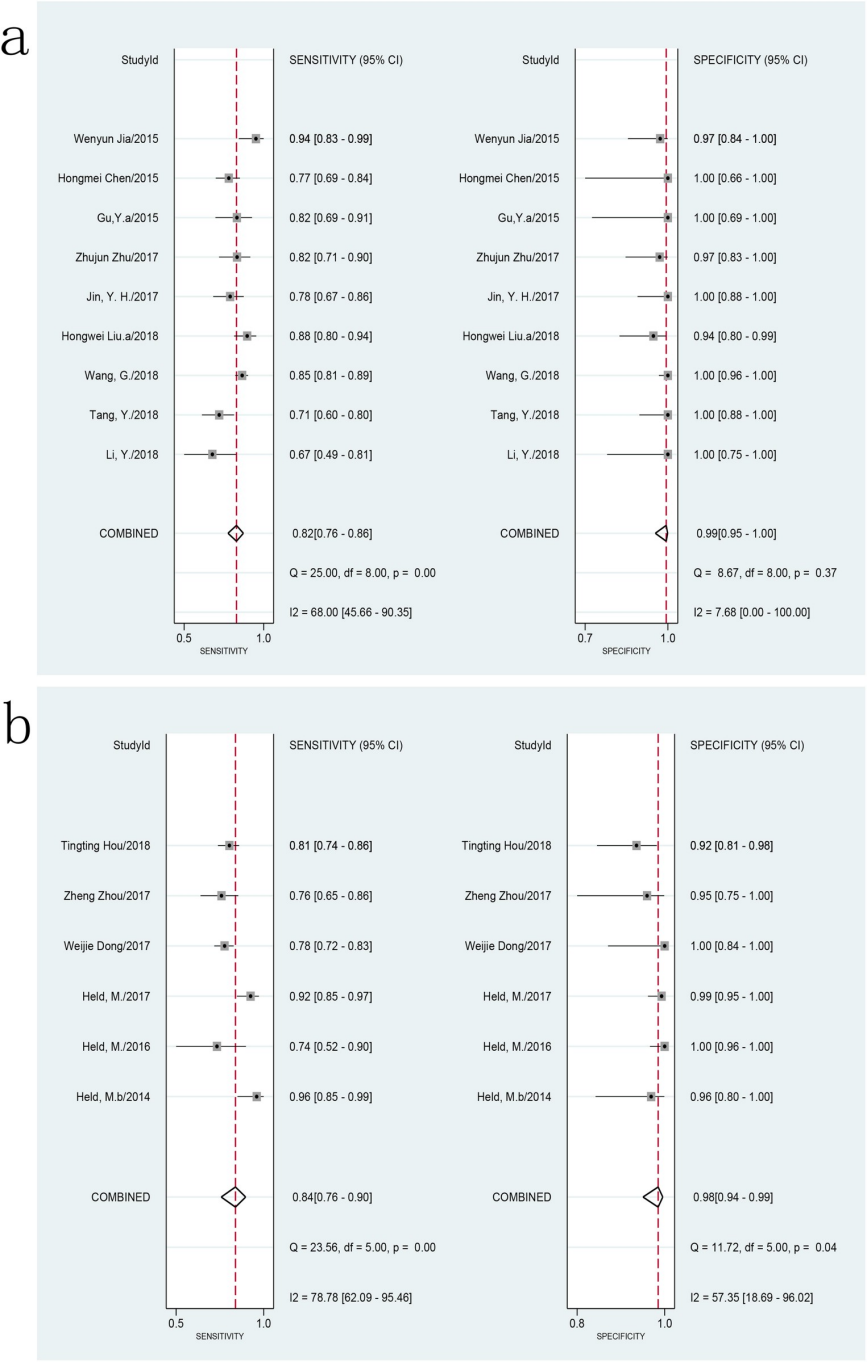
Forest plot of Xpert sensitivity and specificity for tuberculosis detection in BJTB vs. composite reference standard. **a: pus samples. b: tissue samples.** BJTB: Bone and joint tuberculosis.

We explored the heterogeneity among studies, using subgroup and meta-regression analyses on predefined subgroups of sample types, lesion sites, patient selection methods, decontamination methods, sample conditions, and homogenization methods used in the assay. The sensitivity of Xpert MTB/RIF using pus samples ranged from 71% (95% CI, 60–80) to 94% (95% CI, 83–99) and the specificity ranged from 94% (95% CI, 80–99) to 100% (95% CI, 96–100) compared with the CRS. The pooled sensitivity was 82% (*I*^2^ = 68%; 95% CI, 76–86), and the pooled specificity was 99% (*I*^2^ = 8%; 95% CI, 95–100) (Fig 5a). There was an acceptable level of heterogeneity in the specificity and a substantial level of heterogeneity in the sensitivity among studies of Xpert MTB/RIF assay using pus samples compared to the CRS. The AUC of the SROC was 0.95 (95% CI, 0.93–0.97), suggesting a very good overall diagnostic accuracy. Using tissue samples, the sensitivity ranged from 74% (95% CI, 52–90) to 96% (95% CI, 85–99), and the specificity ranged from 92% (95% CI, 81–98) to 100% (95% CI, 96–100) compared with the CRS. The pooled sensitivity and specificity of Xpert MTB/RIF assay using tissue samples vs. CRS were 84% (*I*^2^ = 79%; 95% CI, 76–90) and 98% (*I*^2^ = 57; 95% CI, 94–99), respectively (Fig 5b). There was a substantial level of heterogeneity among studies of Xpert MTB/RIF assay using tissue samples compared to the CRS. The AUC of the SROC was 0.97 (95% CI, 0.95–0.98). Compared to the CRS, studies with pus and tissue samples showed similar levels of sensitivity (82% vs. 84%, P > 0.05) and specificity (98% vs. 99%, P > 0.05), respectively. Only two studies reported separate data regarding joint fluid vs. CRS, and meta-analysis of this specimen type was not performed. When culture results were used as the reference standard, separate data using pus, tissue, and joint fluid were limited, and meta-analysis could not be performed.

**Fig 6:**
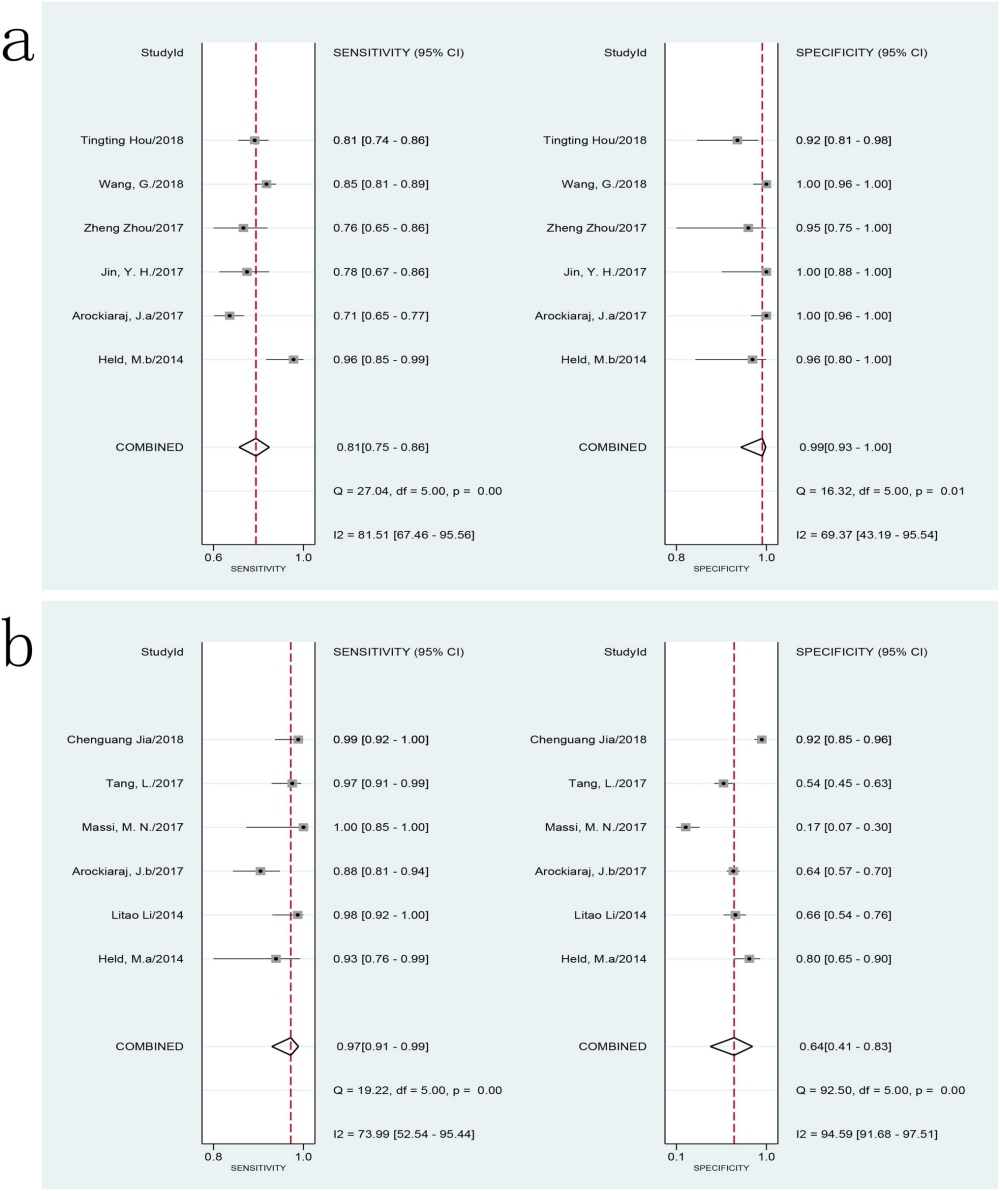
Forest plot of Xpert sensitivity and specificity for tuberculosis detection in BJTB for spinal tuberculosis. **a: composite reference standard. b: culture reference standard.** BJTB: Bone and joint tuberculosis.

For spinal tuberculosis, the sensitivity ranged from 71% (95% CI, 65–77) to 96% (95% CI, 85–99), and the specificity ranged from 92% (95% CI, 81–98) to 100% (95% CI, 96–100) compared with the CRS. The pooled sensitivity and specificity of Xpert MTB/RIF assay for spinal tuberculosis against the CRS were 81% (*I*^2^ = 86%; 95% CI, 75–86) and 99% (*I*^2^ = 69%; 95% CI, 93–100), respectively (Fig 6a). There was a substantial level of heterogeneity among studies. The AUC of the SROC was 0.93 (95% CI, 0.90–0.95). When compared to culture, the sensitivity ranged from 88% (95% CI, 81–94) to 100% (95% CI, 85–100), and the specificity ranged from 17% (95% CI, 7–30) to 92% (95% CI, 85–96). The pooled sensitivity and specificity of Xpert MTB/RIF assay for spinal tuberculosis against culture were 97% (*I*^2^ = 74%; 95% CI, 91–99) and 64% (*I*^2^ = 95%; 95% CI, 41–83) (Fig 6b). The heterogeneity among studies was also substantial. The AUC of the SROC was 0.96 (95% CI, 0.94–0.97). A meta-analysis could not be performed due to limited data on other bone tuberculosis sites.

Meta-regression analysis showed that patient selection method (convenience or consecutive) might affect the sensitivity of the Xpert MTB/RIF assay (80% vs. 83%, meta-regression P < 0.01) but had no effect on its specificity (99% vs. 99%, meta-regression P > 0.05) when compared to the CRS. When compared to culture, the patient selection method did not have any effect on the sensitivity and specificity of the Xpert MTB/RIF assay (meta-regression P > 0.05). Meta-regression analysis showed that decontamination method (with or without N-acetyl-L-cysteine / sodium hydroxide), sample condition (fresh or frozen), method of homogenization (mechanical or otherwise) did not have any effect on the sensitivity and specificity of the Xpert MTB/RIF assay (meta-regression P > 0.05), compared to that of the CRS and culture. These factors were, therefore, obviously not a source of heterogeneity among the studies.

## Discussion

The diagnosis of BJTB, just like that of other forms of EPTB, is very challenging, due to its paucibacillary nature [48]. The delayed diagnosis and treatment of BJTB can lead to serious complications, such as joint destruction, contractures in large joints, growth arrest, and neurological impairment in spinal tuberculosis, resulting in long-term morbidity and disability [36]. Invasive examination is a necessary diagnostic step in most cases. Puncture and biopsy of lesions are the most common invasive procedures for EPTB; the same applies to BJTB. Due to the deep location, obstruction of the bony structure, limited volume of the puncture specimen, the atypical nature of the puncture site, and low bacterial content of the specimen, it is still difficult to diagnose BJTB by pathological and cultural examination of the puncture specimen [39]. This causes BJTB to be often misdiagnosed or missed, leading to inappropriate treatment and adverse prognoses among patients. Therefore, in order to reduce the deformity and disability rate of BJTB, rapid and effective diagnostic methods are needed to detect BJTB early.

Nucleic acid tests such as the loop-mediated isothermal amplification assay, as a fast and efficient detection method, has been widely used in the diagnosis of tuberculosis [49] and demonstrated good diagnostic efficacy in the detection of EPTB [50]. The Xpert MTB/RIF assay, a rapid and automated real-time nucleic acid amplification test is currently one of the most commonly used in the diagnosis of tuberculosis. This test can validate the MTB complex DNA within 2 hours and is widely used in the diagnosis of PTB and EPTB. Previous studies have shown that this test has good diagnostic efficacy in the diagnosis of PTB and EPTB [7]. The Xpert MTB/RIF assay has also been recommended by the World Health Organization for the diagnosis of EPTB [51], including lymph node tuberculous and tuberculous meningitis. This test should also be used in the diagnosis of BJTB. Although many studies had reported the application of this test in BJTB, there has been no consensus on the sensitivity and specificity. In a meta-analysis performed by Wen et al., the pooled sensitivity and specificity of Xpert MTB/RIF for BJTB were 81% (95% CI, 78–83) and 83% (95% CI, 80–86), respectively [52]. However, this result was not differentiated according to different reference standards; the number of studies included was relatively small, and the diagnostic efficacy of different types of specimens and different sites of infection was not differentiated. In order to further evaluate the efficacy of this test in the diagnosis of BJTB, we designed this study to determine the accuracy of Xpert MTB/RIF for BJTB.

Our study included 19 studies with comparisons to the CRS and 14 studies with comparisons to culture. The selection of patients included in most studies was not consecutive. The CRS varied among the articles included, which could be the main source of bias among the studies. Publication bias needed to be considered. For intervention studies, publication bias occurs if studies with significant results are more likely to be published than studies with non-significant findings. Regarding diagnostic tests, many studies are conducted without ethical review or study registration, or do not compare tests; hence, it would be problematic to assess publication bias. According to the Cochrane Handbook for Systematic Reviews of Diagnostic Test Accuracy, traditional analytical approaches, such as ‘funnel plots’, may not be appropriate for the assessment of test accuracy [10]. At present, there is no recognized and accepted statistical method for quantifying the potential effect of publication bias in studies of diagnostic accuracy [53]. Therefore, we chose not to carry out a formal assessment of publication bias.

The present study demonstrated that the pooled sensitivity and specificity of Xpert MTB/RIF for BJTB were 81% (95% CI, 77–84) and 99% (95% CI, 97–100) compared to the CRS, 96% (95% CI, 90–98) and 85% (95% CI, 57–96) compared to culture, respectively. As expected, the use of different reference standards led to different sensitivities and specificities, and their ranges were relatively credible. Regardless of the gold standard, Xpert MTB/RIF showed a very good diagnostic accuracy. In the present study, a substantial level of heterogeneity was also observed among the studies. Subgroup analysis revealed that the pooled sensitivity of Xpert MTB/RIF performed on pus samples was 82% (*I*^2^ = 68%; 95% CI, 76–86), and the pooled specificity was 99% (*I*^2^ = 8%; 95% CI, 95–100) compared to the CRS, respectively. The pooled sensitivity and specificity were 84% (*I*^2^ = 79%; 95% CI, 76–90) and 98% (*I*^2^ = 57%; 95% CI, 94–99), respectively, compared to the CRS, when using tissue specimens. The level of heterogeneity within subgroups decreased, suggesting that specimen types might be a source of heterogeneity among studies especially for specificity. The sensitivity and specificity of Xpert MTB/RIF performed using pus specimens was similar to those obtained using tissue specimens compared to the CRS; there was no significant difference. Overall, the diagnostic accuracy of Xpert MTB/RIF for BJTB, using pus and tissue specimens, were found to be similar. This finding was similar to that made in a previous study of lymph node tuberculosis [8]. For spinal tuberculosis, the pooled sensitivity and specificity of Xpert MTB/RIF were 81% (95% CI, 75–86) vs. 97% (95% CI, 91–99) and 99% (95% CI, 93–100) vs. 64% (95% CI, 41–83) compared to CRS and culture, respectively. However, the heterogeneity (such as regards sample type) across studies was substantial; the results needed to be treated with caution. Owing to its paucibacillary nature, for BJTB, culture is an imperfect reference standard. A CRS with multiple evaluation indicators might be a more applicable reference standard. We chose to evaluate the two reference standards separately as it would be more confusing to mix them. As predicted, we found an underestimated level of sensitivity and an overestimated level of specificity when using the CRS as a reference standard. The range for sensitivity and specificity compared with the two references was more plausible. However, the CRS varied across studies in this study. The CRS for most studies included the results of culture (Lowenstein-Jensen and/or BACTEC MGIT 960 culture), except for four studies [20, 31, 32, 38] while that for most studies included the results of histology/cytology, smear microscopy, clinical symptoms, and radiographic features; eight studies included the response to anti-tuberculosis treatment, and none of the studies included other molecular test results. This might be one of the sources of heterogeneity among the studies. The studies also used different culture references. Nine studies only used BACTEC MGIT 960 liquid culture as the reference, one study only used Lowenstein-Jensen solid culture as the reference [34], and four studies used both of them as references [18,22,24,35]. The performances of BACTEC MGIT 960 liquid and Lowenstein-Jensen solid cultures were different [54], which might also be one of the sources of heterogeneity among the studies. Separate data used in the subgroup of different reference standards were limited; thus, further meta-analysis could not be performed.

Meta-regression analysis showed that the patient selection method affected the outcome and was a source of heterogeneity compared to the CRS, probably due to the fact that patients who were not included consecutively were more likely to introduce selection bias. The sample processing of BJTB specimens, such as decontamination, sample condition, and homogenization, varied among studies; however, meta-regression analysis showed that these factors did not affect the outcome and hence were not obvious sources of heterogeneity.

Our meta-analysis had several limitations. This meta-analysis has not been registered online and despite comprehensive searches, some studies may still have been missed, and some studies failed to distinguish specimen types. In addition, some studies used multiple sample types, which may have led to some bias in our results. Additionally, sample processing of BJTB specimens was highly variable among studies, since the assay, designed for respiratory samples, may slightly vary for other specimens. Additionally, the CRS standard for the studies was different. There was substantial heterogeneity among the studies, and the pooled estimates need to be interpreted with caution.

## Conclusions

We observed that the pooled sensitivity and specificity of Xpert MTB/RIF were 81% and 99%, respectively, when compared to the CRS, and 96% and 85%, respectively, when compared to culture. For spinal tuberculosis, the pooled sensitivity and specificity of Xpert MTB/RIF were 81% and 99%, respectively, when compared with the CRS, and 97% and 64%, respectively, when compared with culture. When performed on pus samples, the pooled sensitivity and specificity were 82% and 99% compared with the CRS. When performed on tissue samples, the pooled sensitivity and specificity were 84% and 98% compared with the CRS. The diagnostic efficiencies for different specimen types (pus and tissue) were similar. Xpert MTB/RIF showed a good diagnostic accuracy for BJTB and was not related to the type of specimen.

## Supporting information

S1 Supporting Information. Data analyzed in this study, including search strategy and funding. All data analyzed in this study are included in this published article and its supplementary information file. (ZIP)

S1 File PRISMA checklist. (DOC)
