# Supplementary material for: Diagnostic accuracy of the Xpert MTB/RIF assay for bone and joint tuberculosis: A meta-analysis
Source: PLoS One. 2019 Aug 22;14(8):e0221427. doi: 10.1371/journal.pone.0221427 (PMC6705841; doi:10.1371/journal.pone.0221427)
Supplement: S1 Supporting Information. Data — (ZIP) [file pone.0221427.s001.zip › S1 Supporting Information/Commands for statistical analysis.docx]

midas tp fp fn tn, id(author year) bfor(dss dlor dlr) texts(0.6) ford fors

midas tp fp fn tn, sroc(both)

midas tp fp fn tn, reg(a)
