# Supplementary material for: Diagnostic accuracy of the Xpert MTB/RIF assay for bone and joint tuberculosis: A meta-analysis
Source: PLoS One. 2019 Aug 22;14(8):e0221427. doi: 10.1371/journal.pone.0221427 (PMC6705841; doi:10.1371/journal.pone.0221427)
Supplement: S1 Supporting Information. Data — (ZIP) [file pone.0221427.s001.zip › S1 Supporting Information/List of excluded studies.docx]

Excluded studies:

1. Pai M, O'Brien R. New diagnostics for latent and active Tuberculosis: State of the art and future prospects. Seminars in Respiratory and Critical Care Medicine. 2008;29(5):560-8. doi: 10.1055/s-0028-1085707.

2. Cain KP, Varma JK. You have to find TB to treat TB. International Journal of Tuberculosis and Lung Disease. 2011;15(7):854. doi: 10.5588/ijtld.11.0247.

3. Causse M, Ruiz P, Gutierrez-Aroca JB, Casal M. Comparison of two molecular methods for rapid diagnosis of extrapulmonary tuberculosis. J Clin Microbiol. 2011;49(8):3065-7. Epub 2011/06/10. doi: 10.1128/jcm.00491-11. PubMed PMID: 21653775; PubMed Central PMCID: PMCPmc3147762.

4. Causse M, Ruiz P, Gutiérrez-Aroca JB, Casal M. Comparison of two molecular methods for rapid diagnosis of extrapulmonary tuberculosis. Journal of Clinical Microbiology. 2011;49(8):3065-7. doi: 10.1128/JCM.00491-11.

5. Clemente MG, Palacios J, Penedo A, Jimenez H, Vázquez S, Díaz-Escalada A, et al. Evaluation of the xpert Mtb/rif test for rapid detection of mycobacterium tuberculosis. American journal of respiratory and critical care medicine. 2011;183(1).

6. Hanif SNM, Eldeen HS, Ahmad S, Mokaddas E. GeneXpert® MTB/RIF for rapid detection of Mycobacterium tuberculosis in pulmonary and extra-pulmonary samples. International Journal of Tuberculosis and Lung Disease. 2011;15(9):1274-5. doi: 10.5588/ijtld.11.0394.

7. Hillemann D, Rusch-Gerdes S, Boehme C, Richter E. Rapid molecular detection of extrapulmonary tuberculosis by the automated GeneXpert MTB/RIF system. J Clin Microbiol. 2011;49(4):1202-5. Epub 2011/01/29. doi: 10.1128/jcm.02268-10. PubMed PMID: 21270230; PubMed Central PMCID: PMCPmc3122824.

8. Hillemann D, Rüsch-Gerdes S, Boehme C, Richter E. Rapid molecular detection of extrapulmonary tuberculosis by the automated GeneXpert MTB/RIF system. Journal of clinical microbiology. 2011;49(4):1202‐5. PubMed PMID: CN-01655375.

9. Hillemann D, Rüsch-Gerdes S, Boehme C, Richter E. Rapid molecular detection of extrapulmonary tuberculosis by the automated genexpert MTB/RIF system. Journal of Clinical Microbiology. 2011;49(4):1202-5. doi: 10.1128/JCM.02268-10.

10. Ioannidis P, Papaventsis D, Karabela S, Nikolaou S, Panagi M, Raftopoulou E, et al. Cepheid geneXpert MTB/RIF assay for Mycobacterium tuberculosis detection and rifampin resistance identification in patients with substantial clinical indications of tuberculosis and smear-negative microscopy results. Journal of Clinical Microbiology. 2011;49(8):3068-70. doi: 10.1128/JCM.00718-11.

11. Lawn SD, Nicol MP. Xpert® MTB/RIF assay: Development, evaluation and implementation of a new rapid molecular diagnostic for tuberculosis and rifampicin resistance. Future microbiology. 2011;6(9):1067-82. doi: 10.2217/fmb.11.84.

12. Malbruny B, Le Marrec G, Courageux K, Leclercq R, Cattoir V. Rapid and efficient detection of Mycobacterium tuberculosis in respiratory and non-respiratory samples. International Journal of Tuberculosis and Lung Disease. 2011;15(4):553-5. doi: 10.5588/ijtld.10.0497.

13. Miller MB, Popowitch EB, Backlund MG, Ager EPC. Performance of Xpert MTB/RIF RUO assay and IS6110 real-time PCR for mycobacterium tuberculosis detection in clinical samples. Journal of Clinical Microbiology. 2011;49(10):3458-62. doi: 10.1128/JCM.05212-11.

14. Vadwai V, Boehme C, Nabeta P, Shetty A, Alland D, Rodrigues C. Xpert MTB/RIF: a new pillar in diagnosis of extrapulmonary tuberculosis? Journal of clinical microbiology. 2011;49(7):2540‐5. PubMed PMID: CN-01655406.

15. Vadwai V, Boehme C, Nabeta P, Shetty A, Alland D, Rodrigues C. Xpert MTB/RIF: a new pillar in diagnosis of extrapulmonary tuberculosis? J Clin Microbiol. 2011;49(7):2540-5. Epub 2011/05/20. doi: 10.1128/jcm.02319-10. PubMed PMID: 21593262; PubMed Central PMCID: PMCPmc3147857.

16. Vadwai V, Boehme C, Nabeta P, Shetty A, Alland D, Rodrigues C. Xpert MTB/RIF: A new pillar in diagnosis of extrapulmonary tuberculosis? Journal of Clinical Microbiology. 2011;49(7):2540-5. doi: 10.1128/JCM.02319-10.

17. Zeka AN, Tasbakan S, Cavusoglu C. Evaluation of the GeneXpert MTB/RIF assay for rapid diagnosis of tuberculosis and detection of rifampin resistance in pulmonary and extrapulmonary specimens. Journal of Clinical Microbiology. 2011;49(12):4138-41. doi: 10.1128/JCM.05434-11.

18. Alvarez-Uria G, Azcona JM, Midde M, Naik PK, Reddy S, Reddy R. Rapid Diagnosis of Pulmonary and Extrapulmonary Tuberculosis in HIV-Infected Patients. Comparison of LED Fluorescent Microscopy and the GeneXpert MTB/RIF Assay in a District Hospital in India. Tuberculosis research and treatment. 2012;2012:932862. Epub 2012/09/12. doi: 10.1155/2012/932862. PubMed PMID: 22966426; PubMed Central PMCID: PMCPmc3433122.

19. Brownell R, Metcalfe J, Millman AJ, Miller C, Cattamanchi A. Performance of Xpert MTB/RIF for diagnosis of pulmonary and extra-pulmonary tuberculosis-a systematic review and meta-analysis. American journal of respiratory and critical care medicine. 2012;185.

20. Chang K, Lu W, Wang J, Zhang K, Jia S, Li F, et al. Rapid and effective diagnosis of tuberculosis and rifampicin resistance with Xpert MTB/RIF assay: a meta-analysis. The Journal of infection. 2012;64(6):580-8. Epub 2012/03/03. doi: 10.1016/j.jinf.2012.02.012. PubMed PMID: 22381459.

21. Chang K, Lu W, Wang J, Zhang K, Jia S, Li F, et al. Rapid and effective diagnosis of tuberculosis and rifampicin resistance with Xpert MTB/RIF assay: A meta-analysis. Journal of Infection. 2012;64(6):580-8. doi: 10.1016/j.jinf.2012.02.012.

22. Clemente MG, Palacios J, Penedo A, Alvarez M, Velasco Z, Pando A, et al. Accuracy of the Xpert MTB/RIF test for rapid diagnosis of extrapulmonary tuberculosis. American journal of respiratory and critical care medicine. 2012;185.

23. Dorjee K, Salvo F, Dierberg KL. Xpert® MTB/RIF diagnosed disseminated smear-negative MDR-TB in a sub-district hospital in India. International Journal of Tuberculosis and Lung Disease. 2012;16(11):1560-1. doi: 10.5588/ijtld.12.0404.

24. Drobniewski FA, Nikolayevskyy V, Balabanova Y, Bang D, Papaventsis D. Diagnosis of tuberculosis and drug resistance: What can new tools bring us? International Journal of Tuberculosis and Lung Disease. 2012;16(7):860-70. doi: 10.5588/ijtld.12.0180.

25. Haraka F, Rutaihwa LK, Battegay M, Reither K. Mycobacterium intracellulare infection in non-HIV infected patient in a region with a high burden of tuberculosis. BMJ case reports. 2012. doi: 10.1136/bcr.01.2012.5713.

26. Lawn SD, Zumla AI. Diagnosis of extrapulmonary tuberculosis using the Xpert((R)) MTB/RIF assay. Expert review of anti-infective therapy. 2012;10(6):631-5. Epub 2012/06/28. doi: 10.1586/eri.12.43. PubMed PMID: 22734954; PubMed Central PMCID: PMCPmc3605769.

27. Lawn SD, Zumla AI. Diagnosis of extrapulmonary tuberculosis using the Xpert® MTB/RIF assay. Expert review of anti-infective therapy. 2012;10(6):631-5. doi: 10.1586/eri.12.43.

28. López Ávalos GG, Prado Montes De Oca E. Classic and new diagnostic approaches to childhood tuberculosis. Journal of Tropical Medicine. 2012. doi: 10.1155/2012/818219.

29. Mehta PK, Raj A, Singh N, Khuller GK. Diagnosis of extrapulmonary tuberculosis by PCR. FEMS Immunology and Medical Microbiology. 2012;66(1):20-36. doi: 10.1111/j.1574-695X.2012.00987.x.

30. Moure R, Martin R, Alcaide F. Effectiveness of an integrated real-time PCR method for detection of the Mycobacterium tuberculosis complex in smear-negative extrapulmonary samples in an area of low tuberculosis prevalence. J Clin Microbiol. 2012;50(2):513-5. Epub 2011/12/14. doi: 10.1128/jcm.06467-11. PubMed PMID: 22162564; PubMed Central PMCID: PMCPmc3264142.

31. Moure R, Martín R, Alcaide F. Effectiveness of an integrated real-time PCR method for detection of the Mycobacterium tuberculosis complex in smear-negative extrapulmonary samples in an area of low tuberculosis prevalence. Journal of Clinical Microbiology. 2012;50(2):513-5. doi: 10.1128/JCM.06467-11.

32. Palomino JC. Current developments and future perspectives for TB diagnostics. Future microbiology. 2012;7(1):59-71. doi: 10.2217/fmb.11.133.

33. Perez-Velez CM. Pediatric tuberculosis: New guidelines and recommendations. Current Opinion in Pediatrics. 2012;24(3):319-28. doi: 10.1097/MOP.0b013e32835357c3.

34. Raj A, Singh N. Tuberculous meningitis- an update on diagnostic approaches. International Journal of Research in Pharmaceutical Sciences. 2012;3(2):312-8.

35. Steingart KR, Ramsay A, Dowdy DW, Pai M. Serological tests for the diagnosis of active tuberculosis: Relevance for India. Indian Journal of Medical Research. 2012;135(5):695-702.

36. Suzana S, Shalini B, Rupali P, Venkatesh K, Christopher DJ, Michael JS. Rapid diagnosis of extra pulmonary tuberculosis by automated Xpert MTB/RIF assay. BMC infectious diseases. 2012;12.

37. Swaminathan S, Rekha VVB. Antigen detection as a point-of-care test for TB: The case of lipoarabinomannan. Future microbiology. 2012;7(5):559-64. doi: 10.2217/fmb.12.34.

38. Taylor N, Gaur RL, Baron EJ, Banaei N. Can a simple flotation method lower the limit of detection of Mycobacterium tuberculosis in extrapulmonary samples analyzed by the GeneXpert MTB/RIF Assay? Journal of Clinical Microbiology. 2012;50(7):2272-6. doi: 10.1128/JCM.01012-12.

39. Tortoli E, Russo C, Piersimoni C, Mazzola E, Dal Monte P, Pascarella M. Clinical validation of Xpert MTB/RIF for the diagnosis of extrapulmonary tuberculosis. European respiratory journal. 2012;40(2):442‐7. PubMed PMID: CN-01655403.

40. Tortoli E, Russo C, Piersimoni C, Mazzola E, Dal Monte P, Pascarella M, et al. Clinical validation of Xpert MTB/RIF for the diagnosis of extrapulmonary tuberculosis. The European respiratory journal. 2012;40(2):442-7. Epub 2012/01/14. doi: 10.1183/09031936.00176311. PubMed PMID: 22241741.

41. Tortoli E, Russo C, Piersimoni C, Mazzola E, Dal Monte P, Pascarella M, et al. Clinical validation of Xpert MTB/RIF for the diagnosis of extrapulmonary tuberculosis. European Respiratory Journal. 2012;40(2):442-7. doi: 10.1183/09031936.00176311.

42. Vadwai V, Boehme C, Nabeta P, Shetty A, Rodrigues C. Need to confirm isoniazid susceptibility in xpert MTB/RIF rifampin susceptible cases. Indian Journal of Medical Research. 2012;135(4):560-1.

43. Banada PP, Koshy R, Alland D. Detection of Mycobacterium tuberculosis in blood by use of the Xpert MTB/RIF assay. J Clin Microbiol. 2013;51(7):2317-22. Epub 2013/05/17. doi: 10.1128/jcm.00332-13. PubMed PMID: 23678063; PubMed Central PMCID: PMCPmc3697682.

44. Banada PP, Koshy R, Alland D. Detection of Mycobacterium tuberculosis in blood by use of the Xpert MTB/RIF assay. Journal of Clinical Microbiology. 2013;51(7):2317-22. doi: 10.1128/JCM.00332-13.

45. Christopher DJ, Schumacher SG, Michael JS, Luo R, Balamugesh T, Duraikannan P, et al. Performance of Xpert MTB/RIF on pleural tissue for the diagnosis of pleural tuberculosis. European Respiratory Journal. 2013;42(5):1427-9. doi: 10.1183/09031936.00103213.

46. Dhana AV, Howell P, Sanne I, Spencer D. Identification of Mycobacterium tuberculosis from pericardial fluid using the new Xpert MTB/RIF assay. BMJ case reports. 2013;2013. Epub 2013/08/30. doi: 10.1136/bcr-2013-200615. PubMed PMID: 23986128; PubMed Central PMCID: PMCPmc3762401.

47. Dhana AV, Howell P, Sanne I, Spencer D. Identification of Mycobacterium tuberculosis from pericardial fluid using the new Xpert MTB/RIF assay. BMJ case reports. 2013. doi: 10.1136/bcr-2013-200615.

48. Fenner L, Ballif M, Graber C, Nhandu V, Dusingize JC, Cortes CP, et al. Tuberculosis in Antiretroviral Treatment Programs in Lower Income Countries: Availability and Use of Diagnostics and Screening. PloS one. 2013;8(10). doi: 10.1371/journal.pone.0077697.

49. Garcia-Clemente M, Buchelli-Ramirez H, Pando-Sandoval A, Palacios J, Alvarez-Alvarez C, Gonzalez-Budino T, et al. Factors assciated with patient and health care system delay in the diagnosis of tuberculosis. American journal of respiratory and critical care medicine. 2013;187.

50. Hella JJ, Hiza HC, Mfinanga E, Reither K. Paraspinal abscess secondary to tuberculous spondylitis diagnosed by Xpert MTB/RIF assay in rural Tanzania. BMJ case reports. 2013;2013. Epub 2013/04/13. doi: 10.1136/bcr-2013-009156. PubMed PMID: 23580687; PubMed Central PMCID: PMCPmc3645768.

51. Hella JJ, Hiza HC, Mfinanga E, Reither K. Paraspinal abscess secondary to tuberculous spondylitis diagnosed by Xpert MTB/RIF assay in rural Tanzania. BMJ case reports. 2013. doi: 10.1136/bcr-2013-009156.

52. Lawn SD, Mwaba P, Bates M, Piatek A, Alexander H, Marais BJ, et al. Advances in tuberculosis diagnostics: The Xpert MTB/RIF assay and future prospects for a point-of-care test. The Lancet Infectious Diseases. 2013;13(4):349-61. doi: 10.1016/S1473-3099(13)70008-2.

53. Nicol MP, Whitelaw A, Stevens W. Using xpert MTB/RIF. Current Respiratory Medicine Reviews. 2013;9(3):187-92. doi: 10.2174/1573398X113099990015.

54. Pinto LM, Udwadia ZF. Xpert MTB/RIF and pulmonary tuberculosis: Time to delve deeper? Thorax. 2013;68(11):987-8. doi: 10.1136/thoraxjnl-2013-203885.

55. Ross CL, Jepson A, Wright C, Singanayagam A, Wickremasinghe M, Berry M, et al. Diagnosis of extrapulmonary tuberculosis using the xpert® MTB/RIF assay for endobronchial ultrasound-guided transbronchial needle aspiration (EBUS-TBNA) samples of mediastinal and hilar lymph nodes. American journal of respiratory and critical care medicine. 2013;187.

56. Singh JA, Bhan A, Upshur R. Diagnosis of drug-resistant TB and provision of second-line TB treatment in India: some ethical considerations. Indian journal of medical ethics. 2013;10(2):110-4. Epub 2013/05/24. doi: 10.20529/ijme.2013.033. PubMed PMID: 23697491.

57. Van Rie A, Page-Shipp L, Mellet K, Scott L, Mkhwnazi M, Jong E, et al. Diagnostic accuracy and effectiveness of the Xpert MTB/RIF assay for the diagnosis of HIV-associated lymph node tuberculosis. European journal of clinical microbiology & infectious diseases. 2013;32(11):1409‐15. doi: 10.1007/s10096-013-1890-0. PubMed PMID: CN-00909954.

58. Van Rie A, Page-Shipp L, Mellet K, Scott L, Mkhwnazi M, Jong E, et al. Diagnostic accuracy and effectiveness of the Xpert MTB/RIF assay for the diagnosis of HIV-associated lymph node tuberculosis. European journal of clinical microbiology & infectious diseases : official publication of the European Society of Clinical Microbiology. 2013;32(11):1409-15. Epub 2013/05/11. doi: 10.1007/s10096-013-1890-0. PubMed PMID: 23660698.

59. Van Rie A, Page-Shipp L, Mellet K, Scott L, Mkhwnazi M, Jong E, et al. Diagnostic accuracy and effectiveness of the Xpert MTB/RIF assay for the diagnosis of HIV-associated lymph node tuberculosis. European Journal of Clinical Microbiology and Infectious Diseases. 2013;32(11):1409-15. doi: 10.1007/s10096-013-1890-0.

60. Zmak L, Jankovic M, Jankovic VK. Evaluation of Xpert MTB/RIF assay for rapid molecular diagnosis of tuberculosis in a two-year period in Croatia. International journal of mycobacteriology. 2013;2(3):179-82. doi: 10.1016/j.ijmyco.2013.05.003.

61. Ablanedo-Terrazas Y, Alvarado-de la Barrera C, Hernandez-Juan R, Ruiz-Cruz M, Reyes-Teran G. Xpert MTB/RIF for diagnosis of tuberculous cervical lymphadenitis in HIV-infected patients. The Laryngoscope. 2014;124(6):1382-5. Epub 2013/10/30. doi: 10.1002/lary.24478. PubMed PMID: 24166585.

62. Ablanedo-Terrazas Y, Alvarado-De La Barrera C, Hernández-Juan R, Ruiz-Cruz M, Reyes-Terán G. Xpert MTB/RIF for diagnosis of tuberculous cervical lymphadenitis in HIV-infected patients. The Laryngoscope. 2014;124(6):1382-5. doi: 10.1002/lary.24478.

63. Alnimr AM, Hassan MI. Potential of two nucleic acid amplification assays for quantifying mycobacterial load in respiratory and non-respiratory specimens: A prospective study. Diagnostic Microbiology and Infectious Disease. 2014;78(3):237-41. doi: 10.1016/j.diagmicrobio.2013.11.020.

64. Auld SC, Moore BK, Killam WP, Eng B, Nong K, Pevzner EC, et al. Rollout of Xpert® MTB/RIF in northwest Cambodia for the diagnosis of tuberculosis among PLHA. Public Health Action. 2014;4(4):216-21. doi: 10.5588/pha.14.0082.

65. Coetzee L, Nicol MP, Jacobson R, Schubert PT, Van Helden PD, Warren RM, et al. Rapid diagnosis of pediatric mycobacterial lymphadenitis using fine needle aspiration biopsy. Pediatric Infectious Disease Journal. 2014;33(9):893-6. doi: 10.1097/INF.0000000000000312.

66. Denkinger CM, Schumacher SG, Boehme CC, Dendukuri N, Pai M, Steingart KR. Xpert MTB/RIF assay for the diagnosis of extrapulmonary tuberculosis: a systematic review and meta-analysis. The European respiratory journal. 2014;44(2):435-46. Epub 2014/04/04. doi: 10.1183/09031936.00007814. PubMed PMID: 24696113.

67. Denkinger CM, Schumacher SG, Boehme CC, Dendukuri N, Pai M, Steingart KR. Xpert MTB/RIF assay for the diagnosis of extrapulmonary tuberculosis: A systematic review and meta-analysis. European Respiratory Journal. 2014;44(2):435-46. doi: 10.1183/09031936.00007814.

68. Dhasmana DJ, Ross C, Bradley CJ, Connell DW, George PM, Singanayagam A, et al. Performance of Xpert MTB/RIF in the diagnosis of tuberculous mediastinal lymphadenopathy by endobronchial ultrasound. Annals of the American Thoracic Society. 2014;11(3):392-6. doi: 10.1513/AnnalsATS.201308-250OC.

69. Gupta S, Abimbola T, Date A, Suthar AB, Bennett R, Sangrujee N, et al. Cost-effectiveness of the Three I's for HIV/TB and ART to prevent TB among people living with HIV. The international journal of tuberculosis and lung disease : the official journal of the International Union against Tuberculosis and Lung Disease. 2014;18(10):1159-65. Epub 2014/09/14. doi: 10.5588/ijtld.13.0571. PubMed PMID: 25216828; PubMed Central PMCID: PMCPmc4886505.

70. Hanrahan CF, Shah M. Economic challenges associated with tuberculosis diagnostic development. Expert Review of Pharmacoeconomics and Outcomes Research. 2014;14(4):499-510. doi: 10.1586/14737167.2014.914438.

71. Held M, Laubscher M, Zar HJ, Dunn RN. GeneXpert polymerase chain reaction for spinal tuberculosis. Bone and joint journal. 2014;96B(10):1366‐9. PubMed PMID: CN-01655373.

72. Held M, Laubscher M, Zar HJ, Dunn RN. GeneXpert polymerase chain reaction for spinal tuberculosis. Bone and Joint Journal. 2014;96B(10):1366-9. doi: 10.1302/0301-620X.96B10.34048.

73. Houston A, MacAllan DC. Extrapulmonary tuberculosis. Medicine (United Kingdom). 2014;42(1):18-22. doi: 10.1016/j.mpmed.2013.10.008.

74. Kim SH. Diagnosis and treatment of extrapulmonary tuberculosis. Journal of the Korean Medical Association. 2014;57(1):34-40. doi: 10.5124/jkma.2014.57.1.34.

75. Lorent N, Choun K, Thai S, Rigouts L, Lynen L. Active tuberculosis screening of close contacts among the urban poor: A Cambodian experience. International Journal of Tuberculosis and Lung Disease. 2014;18(10):1259-60. doi: 10.5588/ijtld.14.0420.

76. Maynard-Smith L, Larke N, Peters JA, Lawn SD. Diagnostic accuracy of the Xpert MTB/RIF assay for extrapulmonary and pulmonary tuberculosis when testing non-respiratory samples: A systematic review. BMC infectious diseases. 2014;14(1). doi: 10.1186/s12879-014-0709-7.

77. Norbis L, Alagna R, Tortoli E, Codecasa LR, Migliori GB, Cirillo DM. Challenges and perspectives in the diagnosis of extrapulmonary tuberculosis. Expert review of anti-infective therapy. 2014;12(5):633-47. doi: 10.1586/14787210.2014.899900.

78. Oniankitan O, Fianyo E, Kakpovi K, Agoda-Koussema LK, Mijiyawa M. Sacrum Pott's disease: A rare location of spine tuberculosis. Egyptian Rheumatologist. 2014;36(4):209-11. doi: 10.1016/j.ejr.2014.02.001.

79. Ozkutuk N, Surucuoglu S. Evaluation of the Xpert MTB/RIF assay for the diagnosis of pulmonary and extrapulmonary tuberculosis in an intermediate-prevalence setting. Mikrobiyoloji bulteni. 2014;48(2):223‐32. PubMed PMID: CN-01655386.

80. Ozkutuk N, Surucuoglu S. [Evaluation of the Xpert MTB/RIF assay for the diagnosis of pulmonary and extrapulmonary tuberculosis in an intermediate-prevalence setting]. Mikrobiyol Bul. 2014;48(2):223-32. Epub 2014/05/14. PubMed PMID: 24819260.

81. Özkütük N, Sürücüog̈lu S. Evaluation of the Xpert MTB/RIF assay for the diagnosis of pulmonary and extrapulmonary tuberculosis in an intermediate-prevalence setting. Mikrobiyoloji Bulteni. 2014;48(2):223-32. doi: 10.5578/mb.7456.

82. Patil N, Saba H, Marco A, Samant R, Mukasa L. Initial experience with GeneXpert MTB/RIF assay in the Arkansas Tuberculosis Control Program. The Australasian medical journal. 2014;7(5):203-7. Epub 2014/06/20. doi: 10.4066/amj.2014.1905. PubMed PMID: 24944716; PubMed Central PMCID: PMCPmc4052441.

83. Patil N, Saba H, Marco A, Samant R, Mukasa L. Initial experience with GeneXpert MTB/RIF assay in the Arkansas tuberculosis control program. Australasian Medical Journal. 2014;7(5):203-7. doi: 10.4066/amj.2014.1905.

84. Scott LE, Beylis N, Nicol M, Nkuna G, Molapo S, Berrie L. Diagnostic accuracy of Xpert MTB/RIF for extrapulmonary tuberculosis specimens: establishing a laboratory testing algorithm for South Africa. Journal of clinical microbiology. 2014;52(6):1818‐23. PubMed PMID: CN-01655397.

85. Scott LE, Beylis N, Nicol M, Nkuna G, Molapo S, Berrie L, et al. Diagnostic accuracy of Xpert MTB/RIF for extrapulmonary tuberculosis specimens: establishing a laboratory testing algorithm for South Africa. J Clin Microbiol. 2014;52(6):1818-23. Epub 2014/03/14. doi: 10.1128/jcm.03553-13. PubMed PMID: 24622091; PubMed Central PMCID: PMCPmc4042800.

86. Scott LE, Beylis N, Nicol M, Nkuna G, Molapo S, Berrie L, et al. Diagnostic accuracy of xpert MTB/RIF for extrapulmonary tuberculosis specimens: Establishing a laboratory testing algorithm for South Africa. Journal of Clinical Microbiology. 2014;52(6):1818-23. doi: 10.1128/JCM.03553-13.

87. Scott LE, Beylis N, Nicol M, Nkuna T, Molapo S, Berrie L, et al. Diagnosing extra pulmonary tuberculosis using Xpert MTB/RIF: A laboratory algorithm. Topics in Antiviral Medicine. 2014;22:424.

88. Scott LE, Beylis N, Nicol M, Nkuna T, Molapo S, Berrie L, et al. Diagnosing Extra Pulmonary Tuberculosis Using Xpert MTB/RIF: A Laboratory Algorithm. Topics in Antiviral Medicine. 2014;22(e-1):424.

89. Shah M, Hanrahan C, Wang ZY, Steingart KR, Lawn SD, Denkinger C, et al. Urine lateral flow lipoarabinomannan assay for diagnosing active tuberculosis in adults living with HIV. Cochrane Database of Systematic Reviews. 2014;2014(12). doi: 10.1002/14651858.CD011420.

90. Sharma SK, Kohli M, Chaubey J, Yadav RN, Sharma R, Singh BK. Evaluation of Xpert MTB/RIF assay performance in diagnosing extrapulmonary tuberculosis among adults in a tertiary care centre in India. European respiratory journal. 2014;44(4):1090‐3. PubMed PMID: CN-01655398.

91. Sharma SK, Kohli M, Chaubey J, Yadav RN, Sharma R, Singh BK, et al. Evaluation of Xpert MTB/RIF assay performance in diagnosing extrapulmonary tuberculosis among adults in a tertiary care centre in India. The European respiratory journal. 2014;44(4):1090-3. Epub 2014/07/27. doi: 10.1183/09031936.00059014. PubMed PMID: 25063241.

92. Sharma SK, Kohli M, Chaubey J, Yadav RN, Sharma R, Singh BK, et al. Evaluation of Xpert MTB/RIF assay performance in diagnosing extrapulmonary tuberculosis among adults in a tertiary care centre in India. European Respiratory Journal. 2014;44(4):1090-3. doi: 10.1183/09031936.00059014.

93. Singh V. Management of MDR TB. Pediatric Pulmonology. 2014;49:S38-S9. doi: 10.1002/ppul.23066.

94. Tebruegge M, Ritz N, Koetz K, Noguera-Julian A, Seddon JA, Welch SB, et al. Availability and use of molecular microbiological and immunological tests for the diagnosis of tuberculosis in europe. PloS one. 2014;9(6):e99129. Epub 2014/06/13. doi: 10.1371/journal.pone.0099129. PubMed PMID: 24922084; PubMed Central PMCID: PMCPmc4055680.

95. Theron G, Peter J, Calligaro G, Meldau R, Hanrahan C, Khalfey H, et al. Determinants of PCR performance (Xpert MTB/RIF), including bacterial load and inhibition, for TB diagnosis using specimens from different body compartments. Scientific reports. 2014;4:5658. Epub 2014/07/12. doi: 10.1038/srep05658. PubMed PMID: 25014250; PubMed Central PMCID: PMCPmc5375978.

96. Tikly M. Chronic bacterial and fungal arthritides. Rheumatology (United Kingdom). 2014;53:i11. doi: 10.1093/rheumatology/keu060.001.

97. Trajman A, da Silva Santos Kleiz de Oliveira EF, Bastos ML, Belo Neto E, Silva EM, da Silva Lourenco MC, et al. Accuracy of polimerase chain reaction for the diagnosis of pleural tuberculosis. Respiratory medicine. 2014;108(6):918-23. Epub 2014/05/08. doi: 10.1016/j.rmed.2014.04.007. PubMed PMID: 24803060.

98. Trajman A, Da Silva Santos Kleiz De Oliveira EF, Bastos ML, Belo Neto E, Silva EM, Da Silva Lourenço MC, et al. Accuracy of polimerase chain reaction for the diagnosis of pleural tuberculosis. Respiratory medicine. 2014;108(6):918-23. doi: 10.1016/j.rmed.2014.04.007.

99. Williams CML, Cheah ESG, Malkin J, Patel H, Otu J, Mlaga K, et al. Face mask sampling for the detection of Mycobacterium tuberculosis in expelled aerosols. PloS one. 2014;9(8). doi: 10.1371/journal.pone.0104921.

100. Bahr NC, Tugume L, Rajasingham R, Kiggundu R, Williams DA, Morawski B, et al. Improved diagnostic sensitivity for tuberculous meningitis with Xpert® MTB/RIF of centrifuged CSF. International Journal of Tuberculosis and Lung Disease. 2015;19(10):1209-15. doi: 10.5588/ijtld.15.0253.

101. Bates M, Mudenda V, Shibemba A, Kaluwaji J, Tembo J, Kabwe M, et al. Burden of tuberculosis at post mortem in inpatients at a tertiary referral centre in sub-Saharan Africa: a prospective descriptive autopsy study. The Lancet Infectious diseases. 2015;15(5):544-51. Epub 2015/03/15. doi: 10.1016/s1473-3099(15)70058-7. PubMed PMID: 25765217.

102. Bates M, Mudenda V, Shibemba A, Kaluwaji J, Tembo J, Kabwe M, et al. Burden of tuberculosis at post mortem in inpatients at a tertiary referral centre in sub-Saharan Africa: A prospective descriptive autopsy study. The Lancet Infectious Diseases. 2015;15(5):544-51. doi: 10.1016/S1473-3099(15)70058-7.

103. Celik C, Gozel MG, Bakici MZ, Berk S, Ozsahin SL, Gulturk E. Applicability of Xpert MTB/RIF assay for routine diagnosis of tuberculosis: a four-year single-center experience. Turkish journal of medical sciences. 2015;45(6):1329-34. Epub 2016/01/19. PubMed PMID: 26775391.

104. Çelik C, Gözel MG, Bakici MZ, Berk S, Özşahin SL, Gültürk E. Applicability of Xpert MTB/RIF assay for routine diagnosis of tuberculosis: A four-year single-center experience. Turkish journal of medical sciences. 2015;45(6):1329-34. doi: 10.3906/sag-1407-56.

105. Chiang SS, Swanson DS, Starke JR. New Diagnostics for Childhood Tuberculosis. Infectious Disease Clinics of North America. 2015;29(3):477-502. doi: 10.1016/j.idc.2015.05.011.

106. Cox JA, Lukande RL, Kalungi S, Van Marck E, Lammens M, Van de Vijver K, et al. Accuracy of Lipoarabinomannan and Xpert MTB/RIF Testing in Cerebrospinal Fluid To Diagnose Tuberculous Meningitis in an Autopsy Cohort of HIV-Infected Adults. J Clin Microbiol. 2015;53(8):2667-73. Epub 2015/06/13. doi: 10.1128/jcm.00624-15. PubMed PMID: 26063865; PubMed Central PMCID: PMCPmc4508395.

107. Dara M, Acosta CD, Melchers NV, Al-Darraji HA, Chorgoliani D, Reyes H, et al. Tuberculosis control in prisons: current situation and research gaps. International journal of infectious diseases : IJID : official publication of the International Society for Infectious Diseases. 2015;32:111-7. Epub 2015/03/27. doi: 10.1016/j.ijid.2014.12.029. PubMed PMID: 25809766.

108. Dara M, Acosta CD, Melchers NVSV, Al-Darraji HAA, Chorgoliani D, Reyes H, et al. Tuberculosis control in prisons: Current situation and research gaps. International Journal of Infectious Diseases. 2015;32:111-7. doi: 10.1016/j.ijid.2014.12.029.

109. Dorman S. Advances in the diagnosis of tuberculosis: Current status and future prospects. International Journal of Tuberculosis and Lung Disease. 2015;19(5):504-16. doi: 10.5588/ijtld.15.0048.

110. Fianyo E, Oniankitan O, Agoda-Koussema LK, Koffi-Tessio VES, Mijiyawa M. White tumor of the wrist: A rare localization of tuberculosis. Egyptian Rheumatologist. 2015;37(1):45-7. doi: 10.1016/j.ejr.2014.05.003.

111. Ghiasi M, Pande T, Pai M. Advances in Tuberculosis Diagnostics. Current Tropical Medicine Reports. 2015;2(2):54-61. doi: 10.1007/s40475-015-0043-1.

112. Gu Y, Wang G, Dong W, Li Y, Ma Y, Shang Y. Xpert MTB/RIF and GenoType MTBDRplus assays for the rapid diagnosis of bone and joint tuberculosis. International journal of infectious diseases. 2015;36:27‐30. PubMed PMID: CN-01655371.

113. Gu Y, Wang G, Dong W, Li Y, Ma Y, Shang Y, et al. Xpert MTB/RIF and GenoType MTBDRplus assays for the rapid diagnosis of bone and joint tuberculosis. International Journal of Infectious Diseases. 2015;36:27-30. doi: 10.1016/j.ijid.2015.05.014.

114. Haraka F, Glass TR, Sikalengo G, Gamell A, Ntamatungiro A, Hatz C, et al. A bundle of services increased ascertainment of tuberculosis among HIV-infected individuals enrolled in a HIV cohort in rural sub-Saharan Africa. PloS one. 2015;10(4). doi: 10.1371/journal.pone.0123275.

115. Iram S, Zeenat A, Hussain S, Yusuf NW, Aslam M. Rapid diagnosis of tuberculosis using Xpert MTB/RIF assay - report from a developing country. Pakistan Journal of Medical Sciences. 2015;31(1). doi: 10.12669/pjms.311.6970.

116. Kim YW, Kwak N, Seong MW, Kim EC, Yoo CG, Kim YW. Accuracy of the Xpert(R) MTB/RIF assay for the diagnosis of extra-pulmonary tuberculosis in South Korea. International journal of tuberculosis and lung disease. 2015;19(1):81‐6. PubMed PMID: CN-01655378.

117. Kim YW, Kwak N, Seong MW, Kim EC, Yoo CG, Kim YW, et al. Accuracy of the Xpert® MTB/RIF assay for the diagnosis of extrapulmonary tuberculosis IN South Korea. International Journal of Tuberculosis and Lung Disease. 2015;19(1):81-6. doi: 10.5588/ijtld.14.0500.

118. Kumar K, Abubakar I. Clinical implications of the global multidrug-resistant tuberculosis epidemic. Clinical Medicine, Journal of the Royal College of Physicians of London. 2015;15:s37-s42. doi: 10.7861/clinmedicine.15-6-s37.

119. Lawn SD. Advances in diagnostic assays for tuberculosis. Cold Spring Harbor Perspectives in Medicine. 2015;5(12). doi: 10.1101/cshperspect.a017806.

120. McNerney R, Cunningham J, Hepple P, Zumla A. New tuberculosis diagnostics and rollout. International Journal of Infectious Diseases. 2015;32:81-6. doi: 10.1016/j.ijid.2015.01.012.

121. Mokaddas E, Ahmad S, Eldeen HS, Al-Mutairi N. Discordance between Xpert MTB/RIF assay and bactec MGIT 960 culture system for detection of rifampin-resistant Mycobacterium tuberculosis isolates in a country with a low tuberculosis (TB) incidence. Journal of Clinical Microbiology. 2015;53(4):1351-4. doi: 10.1128/JCM.03412-14.

122. Montales MT, Chaudhury A, Beebe A, Patil S, Patil N. HIV-Associated TB Syndemic: A Growing Clinical Challenge Worldwide. Frontiers in public health. 2015;3:281. Epub 2016/01/19. doi: 10.3389/fpubh.2015.00281. PubMed PMID: 26779470; PubMed Central PMCID: PMCPmc4688350.

123. Nyirenda OM, Goswami J, Mungwira RG, Divala TH, Kanjala M, Muwalo F, et al. Tuberculosis disease among HIV positive adults on antiretroviral therapy in Malawi. American Journal of Tropical Medicine and Hygiene. 2015;93(4):146.

124. O'Connor BD, Woltmann G, Patel H, Turapov O, Haldar P, Mukamolova GV. Can resuscitation-promoting factors be used to improve culture rates of extra-pulmonary tuberculosis? International Journal of Tuberculosis and Lung Disease. 2015;19(12):1556-7. doi: 10.5588/ijtld.15.0682.

125. O'Grady J, Kik SV, Ferrara G. Extra-pulmonary tuberculosis and Xpert(R) MTB/RIF: all about meta-analyses? The international journal of tuberculosis and lung disease : the official journal of the International Union against Tuberculosis and Lung Disease. 2015;19(3):254. Epub 2015/02/17. doi: 10.5588/ijtld.15.0040. PubMed PMID: 25686127.

126. O'Grady J, Kik SV, Ferrara G. Extra-pulmonary tuberculosis and Xpert® MTB/RIF: All about meta-analyses? International Journal of Tuberculosis and Lung Disease. 2015;19(3):254. doi: 10.5588/ijtld.15.0040.

127. Page AL, Ardizzoni E, Lassovsky M, Kirubi B, Bichkova D, Pedrotta A, et al. Routine use of Xpert MTB/RIF in areas with different prevalences of HIV and drug-resistant tuberculosis. International Journal of Tuberculosis and Lung Disease. 2015;19(9):1078-83. doi: 10.5588/ijtld.14.0951.

128. Penz E, Boffa J, Roberts DJ, Fisher D, Cooper R, Ronksley PE. Diagnostic accuracy of the Xpert((R)) MTB/RIF assay for extra-pulmonary tuberculosis: a meta-analysis. International journal of tuberculosis and lung disease. 2015;19:278‐84. doi: 10.5588/ijtld.14.0262. PubMed PMID: CN-01050315.

129. Penz E, Boffa J, Roberts DJ, Fisher D, Cooper R, Ronksley PE, et al. Diagnostic accuracy of the Xpert(R) MTB/RIF assay for extra-pulmonary tuberculosis: a meta-analysis. The international journal of tuberculosis and lung disease : the official journal of the International Union against Tuberculosis and Lung Disease. 2015;19(3):278-84, i-iii. Epub 2015/02/17. doi: 10.5588/ijtld.14.0262. PubMed PMID: 25686134.

130. Penz E, Boffa J, Roberts DJ, Fisher D, Cooper R, Ronksley PE, et al. Diagnostic accuracy of the Xpert® MTB/RIF assay for extrapulmonary tuberculosis: A meta-analysis. International Journal of Tuberculosis and Lung Disease. 2015;19(3):278-84. doi: 10.5588/ijtld.14.0262.

131. Petrucci R, Lombardi G, Corsini I, Visciotti F, Pirodda A, Cazzato S, et al. Use of transrenal DNA for the diagnosis of extrapulmonary tuberculosis in children: a case of tubercular otitis media. J Clin Microbiol. 2015;53(1):336-8. Epub 2014/10/24. doi: 10.1128/jcm.02548-14. PubMed PMID: 25339389; PubMed Central PMCID: PMCPmc4290950.

132. Petrucci R, Lombardi G, Corsini I, Visciotti F, Pirodda A, Cazzato S, et al. Use of transrenal DNA for the diagnosis of extrapulmonary tuberculosis in children: A case of tubercular otitis media. Journal of Clinical Microbiology. 2015;53(1):336-8. doi: 10.1128/JCM.02548-14.

133. Qin ZZ, Pai M, Van Gemert W, Sahu S, Ghiasi M, Creswell J. How is Xpert MTB/RIF being implemented in 22 high tuberculosis burden countries? European Respiratory Journal. 2015;45(2):549-54. doi: 10.1183/09031936.00147714.

134. Sanjuan-Jimenez R, Toro-Peinado I, Bermudez P, Colmenero JD, Morata P. Comparative Study of a Real-Time PCR Assay Targeting senX3-regX3 versus Other Molecular Strategies Commonly Used in the Diagnosis of Tuberculosis. PloS one. 2015;10(11):e0143025. Epub 2015/11/26. doi: 10.1371/journal.pone.0143025. PubMed PMID: 26600434; PubMed Central PMCID: PMCPmc4658205.

135. Sanjuan-Jimenez R, Toro-Peinado I, Bermudez P, Colmenero JD, Morata P. Comparative study of a real-time PCR assay targeting senX3-regX3 versus other molecular strategies commonly used in the diagnosis of tuberculosis. PloS one. 2015;10(11). doi: 10.1371/journal.pone.0143025.

136. Schumacher SG, Pai M. Xpert(R) MTB/RIF for extra-pulmonary tuberculosis: time to look beyond accuracy. The international journal of tuberculosis and lung disease : the official journal of the International Union against Tuberculosis and Lung Disease. 2015;19(1):2. Epub 2014/12/19. doi: 10.5588/ijtld.14.0850. PubMed PMID: 25519782.

137. Schumacher SG, Pai M. Xpert® MTB/RIF for extra-pulmonary tuberculosis: Time to look beyond accuracy. International Journal of Tuberculosis and Lung Disease. 2015;19(1):2. doi: 10.5588/ijtld.14.0850.

138. Shrestha P, Paudyal B, Basnyat B. GeneXpert MTB/RIF assay as initial test for diagnosis of tuberculous meningitis. BMJ case reports. 2015;2015. Epub 2015/06/14. doi: 10.1136/bcr-2014-207502. PubMed PMID: 26071438; PubMed Central PMCID: PMCPmc4480084.

139. Sikalengo G, Ramírez A, Battegay M, Furrer H, Tanner M, Hatz C, et al. Diagnosis of tuberculous spondylitis through Xpert MTB/RIF assay in urine in rural Africa. Tropical Medicine and International Health. 2015;20:192. doi: 10.1111/tmi.12574.

140. Tadesse M, Abebe G, Abdissa K, Aragaw D, Abdella K, Bekele A, et al. GeneXpert MTB/RIF Assay for the Diagnosis of Tuberculous Lymphadenitis on Concentrated Fine Needle Aspirates in High Tuberculosis Burden Settings. PloS one. 2015;10(9):e0137471. Epub 2015/09/15. doi: 10.1371/journal.pone.0137471. PubMed PMID: 26366871; PubMed Central PMCID: PMCPmc4569183.

141. Tadesse M, Abebe G, Abdissa K, Aragaw D, Abdella K, Bekele A, et al. GeneXpert MTB/RIF assay for the diagnosis of tuberculous lymphadenitis on concentrated fine needle aspirates in high tuberculosis burden settings. PloS one. 2015;10(9). doi: 10.1371/journal.pone.0137471.

142. Walmsley S. Review: Xpert MTB/RIF assay detects extrapulmonary TB in lymph nodes and CSF, but not pleural fluid. Annals of Internal Medicine. 2015;162(4):JC11. doi: 10.7326/ACPJC-2015-162-4-011.

143. 陈红梅, 李雪莲, 高孟秋, 范俊, 张立群. 利福平耐药实时荧光定量核酸扩增检测技术在骨关节结核的诊断价值. 中国临床医生杂志. 2015;43(10):24-6.

144. 贾文韫, 李元. Xpert MTB/RIF在骨关节结核患者快速诊断中的应用. 中国脊柱脊髓杂志. 2015;25(03):208-12.

145. 王桂荣, 谷蕴婷, 董伟杰, 姜广路, 李云絮, 马异峰, et al., editors. Xpert MTB/RIF和GenoType MTBDRplus技术快速诊断骨关节结核及其耐药性的研究. 第三届骨关节结核临床诊断与治疗进展及其规范化专题研讨会; 2015; 中国山东青岛.

146. Ben Ameur S, Smaoui S, Kamoun F, Chabchoub I, Kamoun T, Messaadi F, et al. [Unilateral to bilateral pleurisy: Pleural tuberculosis?]. Archives de pediatrie : organe officiel de la Societe francaise de pediatrie. 2016;23(4):385-8. Epub 2016/02/29. doi: 10.1016/j.arcped.2016.01.014. PubMed PMID: 26922570.

147. Ben Ameur S, Smaoui S, Kamoun F, Chabchoub I, Kamoun T, Messaadi F, et al. Unilateral to bilateral pleurisy: Pleural tuberculosis? Archives de Pediatrie. 2016;23(4):385-8. doi: 10.1016/j.arcped.2016.01.014.

148. Chen ZF, Lao HL, Li XH, Wang J, Chen Q, Wang ZX, et al. [Experimental study of GeneXpert((R)) system in the diagnosis of extra-pulmonary tuberculosis]. Zhonghua jie he he hu xi za zhi = Zhonghua jiehe he huxi zazhi = Chinese journal of tuberculosis and respiratory diseases. 2016;39(7):529-33. Epub 2016/07/20. doi: 10.3760/cma.j.issn.1001-0939.2016.07.008. PubMed PMID: 27430924.

149. Chen ZF, Lao HL, Li XH, Wang J, Chen Q, Wang ZX, et al. Experimental study of GeneXpert(®) system in the diagnosis of extra-pulmonary tuberculosis. Zhonghua jie he he hu xi za zhi = Zhonghua jiehe he huxi zazhi = Chinese journal of tuberculosis and respiratory diseases. 2016;39(7):529-33. doi: 10.3760/cma.j.issn.1001-0939.2016.07.008.

150. Devonshire AS, O'Sullivan DM, Honeyborne I, Jones G, Karczmarczyk M, Pavsic J, et al. The use of digital PCR to improve the application of quantitative molecular diagnostic methods for tuberculosis. BMC infectious diseases. 2016;16:366. Epub 2016/08/05. doi: 10.1186/s12879-016-1696-7. PubMed PMID: 27487852; PubMed Central PMCID: PMCPmc4971652.

151. Diallo AB, Kollo AI, Camara M, Lo S, Ossoga GW, Mbow M. Performance of GeneXpert MTB / RIF in the diagnosis of extrapulmonary tuberculosis in Dakar: 2010-2015. Pan african medical journal. 2016;25:129. doi: 10.11604/pamj.2016.25.129.10065. PubMed PMID: CN-01655366.

152. Diallo AB, Kollo AI, Camara M, Lo S, Ossoga GW, Mbow M, et al. [Performance of GeneXpert MTB / RIF((R)) in the diagnosis of extrapulmonary tuberculosis in Dakar: 2010-2015]. The Pan African medical journal. 2016;25:129. Epub 2017/03/16. doi: 10.11604/pamj.2016.25.129.10065. PubMed PMID: 28292091; PubMed Central PMCID: PMCPmc5325514.

153. Diallo AB, Kollo AI, Camara M, Lo S, Ossoga GW, Mbow M, et al. Performance of GeneXpert MTB / RIF® in the diagnosis of extrapulmonary tuberculosis in Dakar: 2010-2015. Pan African Medical Journal. 2016;25. doi: 10.11604/pamj.2016.25.129.10065.

154. Diop SA, Massaly A, Ka D, Manga NM, Fortes-Déguénonvo L, Ndour CT, et al. Use of GeneXpert test for the diagnosis of tuberculosis in the department of infectious diseases of FANN University Hospital. Pan African Medical Journal. 2016;23. doi: 10.11604/pamj.2016.23.244.7442.

155. Fanosie A, Gelaw B, Tessema B, Tesfay W, Admasu A, Yitayew G. Mycobacterium tuberculosis Complex and HIV Co-Infection among Extrapulmonary Tuberculosis Suspected Cases at the University of Gondar Hospital, Northwestern Ethiopia. PloS one. 2016;11(3):e0150646. Epub 2016/03/08. doi: 10.1371/journal.pone.0150646. PubMed PMID: 26950547; PubMed Central PMCID: PMCPmc4780813.

156. Fanosie A, Gelaw B, Tessema B, Tesfay W, Admasu A, Yitayew G. Mycobacterium Tuberculosis complex and HIV co-infection among Extrapulmonary Tuberculosis suspected cases at the University of Gondar hospital, northwestern Ethiopia. PloS one. 2016;11(3). doi: 10.1371/journal.pone.0150646.

157. Gutierrez C. Benefits and challenges of molecular diagnostics for childhood tuberculosis. International journal of mycobacteriology. 2016;5:S4-S5. doi: 10.1016/j.ijmyco.2016.08.011.

158. Held M, Laubscher M, Mears S, Dix-Peek S, Workman L, Zar H, et al. Diagnostic Accuracy of the Xpert MTB/RIF Assay for Extrapulmonary Tuberculosis in Children With Musculoskeletal Infections. The Pediatric infectious disease journal. 2016;35(11):1165-8. Epub 2016/10/21. doi: 10.1097/inf.0000000000001271. PubMed PMID: 27286562; PubMed Central PMCID: PMCPmc5071124.

159. Held M, Laubscher M, Mears S, Dix-Peek S, Workman L, Zar H, et al. Diagnostic Accuracy of the Xpert MTB/RIF Assay for Extrapulmonary Tuberculosis in Children with Musculoskeletal Infections. Pediatric Infectious Disease Journal. 2016;35(11):1165-8. doi: 10.1097/INF.0000000000001271.

160. Hodkinson B, Osman N, Botha-Scheepers S. HIV Infection and Osteoarticular Tuberculosis: Strange Bedfellows. Case reports in rheumatology. 2016;2016:5718423. Epub 2016/07/02. doi: 10.1155/2016/5718423. PubMed PMID: 27366339; PubMed Central PMCID: PMCPmc4899592.

161. Hosseinipour MC, Bisson GP, Miyahara S, Sun X, Moses A, Riviere C, et al. Empirical tuberculosis therapy versus isoniazid in adult outpatients with advanced HIV initiating antiretroviral therapy (REMEMBER): a multicountry open-label randomised controlled trial. Lancet (London, England). 2016;387(10024):1198-209. Epub 2016/03/31. doi: 10.1016/s0140-6736(16)00546-8. PubMed PMID: 27025337; PubMed Central PMCID: PMCPmc4931281.

162. Hosseinipour MC, Bisson GP, Miyahara S, Sun X, Moses A, Riviere C, et al. Empirical tuberculosis therapy versus isoniazid in adult outpatients with advanced HIV initiating antiretroviral therapy (REMEMBER): A multicountry open-label randomised controlled trial. The Lancet. 2016;387(10024):1198-209. doi: 10.1016/S0140-6736(16)00546-8.

163. Karat AS, Omar T, Von Gottberg A, Tlali M, Chihota VN, Churchyard GJ, et al. Autopsy prevalence of tuberculosis and other potentially treatable infections among adults with advanced HIV enrolled in out-patient care in South Africa. PloS one. 2016;11(11). doi: 10.1371/journal.pone.0166158.

164. Maraba N, Karat AS, McCarthy K, Churchyard GJ, Charalambous S, Kahn K, et al. Verbal autopsy-assigned causes of death among adults being investigated for TB in South Africa. Transactions of the Royal Society of Tropical Medicine and Hygiene. 2016;110(9):510-6. doi: 10.1093/trstmh/trw058.

165. Marouane C, Smaoui S, Kammoun S, Slim L, Messadi-Akrout F. Evaluation of molecular detection of extrapulmonary tuberculosis and resistance to rifampicin with GeneXpert(R) MTB/RIF. Medecine et maladies infectieuses. 2016;46(1):20-4. Epub 2015/12/15. doi: 10.1016/j.medmal.2015.10.012. PubMed PMID: 26654321.

166. Marouane C, Smaoui S, Kammoun S, Slim L, Messadi-Akrout F. Evaluation of molecular detection of extrapulmonary tuberculosis and resistance to rifampicin with GeneXpert® MTB/RIF. Medecine et maladies infectieuses. 2016;46(1):20-4. doi: 10.1016/j.medmal.2015.10.012.

167. Mazzola E, Arosio M, Nava A, Fanti D, Gesu G, Farina C. Performance of real-time PCR Xpert (R)MTB/RIF in diagnosing extrapulmonary tuberculosis. Infezioni in medicina. 2016;24(4):304‐9. PubMed PMID: CN-01655383.

168. Mazzola E, Arosio M, Nava A, Fanti D, Gesu G, Farina C. Performance of real-time PCR Xpert (R)MTB/RIF in diagnosing extrapulmonary tuberculosis. Le infezioni in medicina : rivista periodica di eziologia, epidemiologia, diagnostica, clinica e terapia delle patologie infettive. 2016;24(4):304-9. Epub 2016/12/25. PubMed PMID: 28011966.

169. Mazzola E, Arosio M, Nava A, Fanti D, Gesu G, Farina C. Performance of real-time PCR Xpert ®MTB/RIF in diagnosing extrapulmonary tuberculosis. Infezioni in Medicina. 2016;24(4):304-9.

170. Mokaddas EM, Saadaldeen H, Ahmad S. Comparison of two molecular methods and an automated liquid culture system for the early detection of Mycobacterium tuberculosis from both pulmonary and extrapulmonary specimens in Kuwait. International journal of mycobacteriology. 2016;5:S74-S5. doi: 10.1016/j.ijmyco.2016.09.004.

171. Musoke J, Michel AL. Characteristics of tuberculosis patients and the evaluation of compliance to the national TB management guidelines at clinics in a rural community from Mpumalanga province, South Africa. Southern African Journal of Epidemiology and Infection. 2016;31(4):135-7. doi: 10.1080/23120053.2016.1156879.

172. Nataraj G, Kanade S, Mehta P. Xpert((R)) MTB/RIF for improved case detection of extra-pulmonary TB in a tertiary care setting in urban India. The international journal of tuberculosis and lung disease : the official journal of the International Union against Tuberculosis and Lung Disease. 2016;20(7):890-4. Epub 2016/06/12. doi: 10.5588/ijtld.15.0849. PubMed PMID: 27287640.

173. Nataraj G, Kanade S, Mehta P. XpertW MTB/RIF for improved case detection of extra-pulmonary TB in a tertiary care setting in urban India. International Journal of Tuberculosis and Lung Disease. 2016;20(7):890-4. doi: 10.5588/ijtld.15.0849.

174. Park JS. Issues Related to the Updated 2014 Korean Guidelines for Tuberculosis. Tuberculosis and respiratory diseases. 2016;79(1):1-4. Epub 2016/01/16. doi: 10.4046/trd.2016.79.1.1. PubMed PMID: 26770228; PubMed Central PMCID: PMCPmc4701788.

175. Penata A, Salazar R, Castano T, Bustamante J, Ospina S. Molecular diagnosis of extrapulmonary tuberculosis and sensitivity to rifampicin with an automated real-time method. Biomedica : revista del Instituto Nacional de Salud. 2016;36(0):78-89. Epub 2016/09/14. doi: 10.7705/biomedica.v36i3.3088. PubMed PMID: 27622628.

176. Peñata A, Salazar R, Castaño T, Bustamante J, Ospina S. Molecular diagnosis of extrapulmonary tuberculosis and sensitivity to rifampicin with an automated real-time method. Biomedica : revista del Instituto Nacional de Salud. 2016;36:78-89. doi: 10.7705/biomedica.v36i3.3088.

177. Ramamurthy K, Bhat S, Shenoy S, Rangnekar A. Xpert Mycobacterium tuberculosis/rifampicin assay: A boon in tuberculosis diagnostics. Asian Journal of Pharmaceutical and Clinical Research. 2016;9(5):225-7.

178. Ramjathan P, Reddy N, Mlisana KP. Utility of GeneXpert MTB/RIF assay in the diagnosis of extrapulmonary tuberculosis. Journal of the International AIDS Society. 2016;19:162-3. doi: 10.7448/IAS.19.6.21264.

179. Rathi P, Gambhire P. Abdominal Tuberculosis. The Journal of the Association of Physicians of India. 2016;64(2):38-47. Epub 2016/10/13. PubMed PMID: 27730779.

180. Rathi P, Gambhire P. Abdominal Tuberculosis. The Journal of the Association of Physicians of India. 2016;64(2):38-47.

181. Shah M, Hanrahan C, Wang ZY, Dendukuri N, Lawn SD, Denkinger CM, et al. Lateral flow urine lipoarabinomannan assay for detecting active tuberculosis in HIV‐positive adults. Cochrane Database of Systematic Reviews. 2016;(5). doi: 10.1002/14651858.CD011420.pub2. PubMed PMID: CD011420.

182. Sharma A, Chhabra HS, Mahajan R, Chabra T, Batra S. Magnetic Resonance Imaging and GeneXpert: A Rapid and Accurate Diagnostic Tool for the Management of Tuberculosis of the Spine. Asian spine journal. 2016;10(5):850-6. Epub 2016/10/30. doi: 10.4184/asj.2016.10.5.850. PubMed PMID: 27790312; PubMed Central PMCID: PMCPmc5081319.

183. Sikalengo G, Ramirez A, Faini D, Mwamelo K, Battegay M, Jugheli L, et al. Tuberculous spondylitis diagnosed through Xpert MTB/RIF assay in urine: a case report. BMC infectious diseases. 2016;16(1):514. Epub 2016/09/28. doi: 10.1186/s12879-016-1844-0. PubMed PMID: 27670679; PubMed Central PMCID: PMCPmc5037907.

184. Sikalengo G, Ramirez A, Faini D, Mwamelo K, Battegay M, Jugheli L, et al. Tuberculous spondylitis diagnosed through Xpert MTB/RIF assay in urine: A case report. BMC infectious diseases. 2016;16(1). doi: 10.1186/s12879-016-1844-0.

185. Singh M, Sethi GR, Mantan M, Khanna A, Hanif M. Xpert® MTB/RIF assay for the diagnosis of pulmonary tuberculosis in children. International Journal of Tuberculosis and Lung Disease. 2016;20(6):839-43. doi: 10.5588/ijtld.15.0824.

186. Singh UB, Pandey P, Mehta G, Bhatnagar AK, Mohan A, Goyal V, et al. Genotypic, phenotypic and clinical validation of genexpert in extra-pulmonary and pulmonary tuberculosis in India. PloS one. 2016;11(2). doi: 10.1371/journal.pone.0149258.

187. Soto-Martinez ME. A review of current management and diagnosis criteria of tuberculosis in children. Pediatric Pulmonology. 2016;51:S50-S2. doi: 10.1002/ppul.23455.

188. Suzana S, Ninan MM, Gowri M, Venkatesh K, Rupali P, Michael JS. Xpert MTB/Rif for the diagnosis of extrapulmonary tuberculosis - an experience from a tertiary care centre in South India. Tropical medicine & international health. 2016;21(3):385‐92. PubMed PMID: CN-01655401.

189. Suzana S, Ninan MM, Gowri M, Venkatesh K, Rupali P, Michael JS. Xpert MTB/Rif for the diagnosis of extrapulmonary tuberculosis--an experience from a tertiary care centre in South India. Tropical medicine & international health : TM & IH. 2016;21(3):385-92. Epub 2015/12/17. doi: 10.1111/tmi.12655. PubMed PMID: 26671654.

190. Suzana S, Ninan MM, Gowri M, Venkatesh K, Rupali P, Michael JS. Xpert MTB/Rif for the diagnosis of extrapulmonary tuberculosis - an experience from a tertiary care centre in South India. Tropical Medicine and International Health. 2016;21(3):385-92. doi: 10.1111/tmi.12655.

191. Thakkar K, Ghaisas SM, Singh M. Lymphadenopathy: Differentiation between Tuberculosis and Other Non-Tuberculosis Causes like Follicular Lymphoma. Frontiers in public health. 2016;4:31. Epub 2016/03/05. doi: 10.3389/fpubh.2016.00031. PubMed PMID: 26942176; PubMed Central PMCID: PMCPmc4766275.

192. Tompkins KM, Reimers MA, White BL, Herce ME. Diagnosis of Concurrent Pulmonary Tuberculosis and Tuberculous Otitis Media Confirmed by Xpert MTB/RIF in the United States. Infectious diseases in clinical practice (Baltimore, Md). 2016;24(3):180-2. Epub 2016/06/28. doi: 10.1097/ipc.0000000000000333. PubMed PMID: 27346926; PubMed Central PMCID: PMCPmc4915369.

193. Tompkins KM, Reimers MA, White BL, Herce ME. Diagnosis of concurrent pulmonary tuberculosis and tuberculous otitis media confirmed by Xpert MTB/RIF in the United States. Infectious Diseases in Clinical Practice. 2016;24(3):180-2. doi: 10.1097/IPC.0000000000000333.

194. Walusimbi S, Semitala F, Bwanga F, Haile M, De Costa A, Davis L, et al. Outcomes of a clinical diagnostic algorithm for management of ambulatory smear and Xpert MTB/Rif negative HIV infected patients with presumptive pulmonary TB in Uganda: a prospective study. The Pan African medical journal. 2016;23:154. Epub 2016/06/16. doi: 10.11604/pamj.2016.23.154.7995. PubMed PMID: 27303572; PubMed Central PMCID: PMCPmc4894731.

195. Wei G, Mu J, Wang G, Huo F, Dong L, Li Y, et al. The reliability analysis of Xpert-positive result for smear-negative and culture-negative specimen collected from bone and joint tuberculosis suspects. Journal of thoracic disease. 2016;8(6):1205-9. Epub 2016/06/14. doi: 10.21037/jtd.2016.04.19. PubMed PMID: 27293838; PubMed Central PMCID: PMCPmc4885960.

196. Wei G, Mu J, Wang G, Huo F, Dong L, Li Y, et al. The reliability analysis of Xpert-positive result for smear-negative and culture-negative specimen collected from bone and joint tuberculosis suspects. Journal of thoracic disease. 2016;8(6):1205-9. doi: 10.21037/jtd.2016.04.19.

197. Yuan M, Lyu Y, Chen ST, Cai C, Li Y, Zhang ZG, et al. Evaluation of Xpert MTB/RIF for the Diagnosis of Extrapulmonary Tuberculosis in China. Biomedical and environmental sciences : BES. 2016;29(8):599-602. Epub 2016/09/24. doi: 10.3967/bes2016.080. PubMed PMID: 27660225.

198. Yuan M, Lyu Y, Chen ST, Cai C, Li Y, Zhang ZG, et al. Evaluation of Xpert MTB/RIF for the Diagnosis of Extrapulmonary Tuberculosis in China. Biomedical and environmental sciences : BES. 2016;29(8):599-602.

199. Zhang AM, Li F, Liu XH, Xia L, Lu SH. [Application of Gene Xpert Mycobacterium tuberculosis DNA and resistance to rifampicin assay in the rapid detection of tuberculosis in children]. Zhonghua er ke za zhi = Chinese journal of pediatrics. 2016;54(5):370-4. Epub 2016/05/05. doi: 10.3760/cma.j.issn.0578-1310.2016.05.012. PubMed PMID: 27143080.

200. Zhang AM, Li F, Liu XH, Xia L, Lu SH. Application of Gene Xpert Mycobacterium tuberculosis DNA and resistance to rifampicin assay in the rapid detection of tuberculosis in children. Zhonghua er ke za zhi = Chinese journal of pediatrics. 2016;54(5):370-4. doi: 10.3760/cma.j.issn.0578-1310.2016.05.012.

201. 李祯. Xpert MTB/RIF检测骨关节结核病灶中结核分枝杆菌及利福平耐药性的研究 [硕士]: 山西医科大学; 2016.

202. 李祯, 马远征, 秦世炳. GeneXpert MTB/RIF技术的应用进展及其在骨关节结核诊断中的价值. 中国防痨杂志. 2016;38(4):322-6. doi: 10.3969/j.issn.1000-6621.2016.04.019.

203. 刘荣梅, 高孟秋, 吴晓光, 张立群, 宋艳华, 马丽萍. 利福平耐药实时荧光定量核酸扩增检测技术与γ-干扰素释放试验在关节结核辅助诊断中的价值. 中国医刊. 2016;51(6):70-2. doi: 10.3969/j.issn.1008-1070.2016.06.021.

204. 刘荣梅, 高孟秋, 吴晓光, 张立群, 宋艳华, 马丽萍. 利福平耐药实时荧光定量核酸扩增检测技术与γ-干扰素释放试验在关节结核辅助诊断中的价值. 中国医刊. 2016;51(06):70-2.

205. Al-Azri MS, Manarang J, Al-Mufargi Y. Bacille Calmette-Guerin Vaccine-Induced Tuberculous Hip Osteomyelitis in an Infant: Case report. Sultan Qaboos University medical journal. 2017;17(3):e358-e62. Epub 2017/10/25. doi: 10.18295/squmj.2017.17.03.019. PubMed PMID: 29062564; PubMed Central PMCID: PMCPmc5642371.

206. Al-Azri MS, Manarang J, Al-Mufargi Y. Bacille Calmette-Guérin Vaccine-Induced Tuberculous Hip Osteomyelitis in an Infant: Case report. Sultan Qaboos University medical journal. 2017;17(3):e358-e62. doi: 10.18295/squmj.2017.17.03.019.

207. Altez-Fernandez C, Ortiz V, Mirzazadeh M, Zegarra L, Seas C, Ugarte-Gil C. Diagnostic accuracy of nucleic acid amplification tests (NAATs) in urine for genitourinary tuberculosis: a systematic review and meta-analysis. BMC infectious diseases. 2017;17(1):390. Epub 2017/06/07. doi: 10.1186/s12879-017-2476-8. PubMed PMID: 28583076; PubMed Central PMCID: PMCPmc5460328.

208. Altez-Fernandez C, Ortiz V, Mirzazadeh M, Zegarra L, Seas C, Ugarte-Gil C. Diagnostic accuracy of nucleic acid amplification tests (NAATs) in urine for genitourinary tuberculosis: A systematic review and meta-analysis. BMC infectious diseases. 2017;17(1). doi: 10.1186/s12879-017-2476-8.

209. Arockiaraj J, Michael JS, Amritanand R, David KS, Krishnan V. The role of Xpert MTB/RIF assay in the diagnosis of tubercular spondylodiscitis. European Spine Journal. 2017;26(12):3162-9. doi: 10.1007/s00586-017-5076-9.

210. Babafemi EO, Cherian BP, Banting L, Mills GA, Ngianga K. Effectiveness of real-time polymerase chain reaction assay for the detection of Mycobacterium tuberculosis in pathological samples: A systematic review and meta-analysis. Systematic Reviews. 2017;6(1). doi: 10.1186/s13643-017-0608-2.

211. Bacha JM, Ngo K, Clowes P, Draper HR, Ntinginya EN, DiNardo A, et al. Why being an expert - despite xpert -remains crucial for children in high TB burden settings. BMC infectious diseases. 2017;17(1). doi: 10.1186/s12879-017-2236-9.

212. Chaves W, Buitrago JF, Dueñas A, Bejarano JC. On extrapulmonary tuberculosis. Repertorio de Medicina y Cirugia. 2017;26(2):90-7. doi: 10.1016/j.reper.2017.04.004.

213. Che N, Yang X, Liu Z, Li K, Chen X. Rapid Detection of Cell-Free Mycobacterium tuberculosis DNA in Tuberculous Pleural Effusion. J Clin Microbiol. 2017;55(5):1526-32. Epub 2017/03/10. doi: 10.1128/jcm.02473-16. PubMed PMID: 28275073; PubMed Central PMCID: PMCPmc5405270.

214. Che N, Yang X, Liu Z, Li K, Chen X. Rapid detection of cell-free Mycobacterium tuberculosis DNA in tuberculous pleural effusion. Journal of Clinical Microbiology. 2017;55(5):1526-32. doi: 10.1128/JCM.02473-16.

215. Clouse K, Blevins M, Lindegren ML, Yotebieng M, Nguyen DT, Omondi A, et al. Low implementation of Xpert MTB/RIF among HIV/TB co-infected adults in the International epidemiologic Databases to Evaluate AIDS (IeDEA) program. PloS one. 2017;12(2). doi: 10.1371/journal.pone.0171384.

216. Elhassan MM, Hemeg HA, Elmekki MA, Turkistani KA, Abdul-Aziz AA. Burden of multidrug resistant Mycobacterium tuberculosis among new cases in Al-Madinah Al-Monawarah, Saudi Arabia. Infectious Disorders - Drug Targets. 2017;17(1):14-23. doi: 10.2174/1871526517666161116104242.

217. Garcia P, Balcells ME, Castillo C, Miranda C, Geoffroy E, Roman JC, et al. [Evaluation of Xpert(R) MTB/RIF technique for Mycobacterium tuberculosis complex detection in extra-respiratory specimens]. Revista chilena de infectologia : organo oficial de la Sociedad Chilena de Infectologia. 2017;34(4):333-9. Epub 2017/11/23. doi: 10.4067/s0716-10182017000400333. PubMed PMID: 29165509.

218. Harinath B. Tuberculosis serodiagnostics: Ban and after. International journal of mycobacteriology. 2017;6(4):323-5. doi: 10.4103/ijmy.ijmy_144_17.

219. Held MFG, Hoppe S, Laubscher M, Mears S, Dix-Peek S, Zar HJ, et al. Epidemiology of Musculoskeletal Tuberculosis in an Area with High Disease Prevalence. Asian spine journal. 2017;11(3):405-11. Epub 2017/07/04. doi: 10.4184/asj.2017.11.3.405. PubMed PMID: 28670408; PubMed Central PMCID: PMCPmc5481595.

220. Hossain MD, Rahim MA, Ahmed JU, Amin MK, Islam S. Usefulness of gene xpert MTB/RIF in the diagnosis of extra-pulmonary tuberculosis. Respirology (Carlton, Vic). 2017;22:134. doi: 10.1111/resp.13207_138.

221. Jain A, Singh PK, Singh U, Kumar V. Initial screening of extra-pulmonary tuberculosis using the Xpert MTB/RIF assay improves case detection rates. The international journal of tuberculosis and lung disease : the official journal of the International Union against Tuberculosis and Lung Disease. 2017;21(4):478-80. Epub 2017/03/13. doi: 10.5588/ijtld.17.0018. PubMed PMID: 28284274.

222. Jain A, Singh PK, Singh U, Kumar V. Initial screening of extra-pulmonary tuberculosis using the Xpert MTB/RIF assay improves case detection rates. International Journal of Tuberculosis and Lung Disease. 2017;21(4):478-80. doi: 10.5588/ijtld.17.0018.

223. Jin YH, Shi SY, Zheng Q, Shen J, Ying XZ, Wang YF. Application value of Xpert MTB/RIF in diagnosis of spinal tuberculosis and detection of rifampin resistance. Zhongguo gu shang = China journal of orthopaedics and traumatology. 2017;30(9):787-91. doi: 10.3969/j.issn.1003-0034.2017.09.002.

224. Jing H, Lu ZM, Deng YF, Gao DC, Li L, Graviss EA. Evaluation of Xpert MTB/RIF in detection of pulmonary and extrapulmonary tuberculosis cases in China. International journal of clinical and experimental pathology. 2017;10(4):4847‐51. PubMed PMID: CN-01655377.

225. Khromova PA, Ogarkov OB, Zhdanova SN, Sinkov VV, Moiseeva EY, Tzyrenova TA, et al. [The detection of highly-transmissible genotypes of agent in clinical samples for prognosis of unfavorable course of tuberculosis.]. Klinicheskaia laboratornaia diagnostika. 2017;62(10):622-7. Epub 2017/01/01. doi: 10.18821/0869-2084-2017-62-10-622-627. PubMed PMID: 30821945.

226. Kohli M, Schiller I, Dendukuri N, Ryan H, Dheda K, Denkinger CM, et al. Xpert® MTB/RIF assay for extrapulmonary tuberculosis and rifampicin resistance. Cochrane Database of Systematic Reviews. 2017;2017(8). doi: 10.1002/14651858.CD012768.

227. Laniado-Laborín R. Diagnosis and treatment of drug-resistant tuberculosis: State of the art. Current Respiratory Medicine Reviews. 2017;13(2):73-81. doi: 10.2174/1573398X13666170926154425.

228. Li Y, Pang Y, Zhang T, Xian X, Wang X, Yang J, et al. Rapid diagnosis of extrapulmonary tuberculosis with Xpert Mycobacterium tuberculosis/rifampicin assay. J Med Microbiol. 2017;66(7):910-4. Epub 2017/07/15. doi: 10.1099/jmm.0.000522. PubMed PMID: 28708053.

229. Li Y, Pang Y, Zhang T, Xian X, Wang X, Yang J, et al. Rapid diagnosis of extrapulmonary tuberculosis with Xpert Mycobacterium tuberculosis/rifampicin assay. Journal of Medical Microbiology. 2017;66(7):910-4. doi: 10.1099/jmm.0.000522.

230. Lombardi G, Di Gregori V, Girometti N, Tadolini M, Bisognin F, Dal Monte P. Diagnosis of smear-negative tuberculosis is greatly improved by Xpert MTB/RIF. PloS one. 2017;12(4):e0176186. Epub 2017/04/22. doi: 10.1371/journal.pone.0176186. PubMed PMID: 28430807; PubMed Central PMCID: PMCPmc5400262.

231. Lombardi G, Di Gregori V, Girometti N, Tadolini M, Bisognin F, Dal Monte P. Diagnosis of smear-negative tuberculosis is greatly improved by Xpert MTB/RIF. PloS one. 2017;12(4). doi: 10.1371/journal.pone.0176186.

232. Lu J, Li H, Dong F, Shi J, Yang H, Han S, et al. The Feasibility of Xpert MTB/RIF Testing to Detect Rifampicin Resistance among Childhood Tuberculosis for Prevalence Surveys in Northern China. BioMed research international. 2017;2017:5857369. Epub 2018/01/24. doi: 10.1155/2017/5857369. PubMed PMID: 29359155; PubMed Central PMCID: PMCPmc5735616.

233. Magnerou AM, Ngounsgoure HFS, Sounga Banzouzi EP, Ndiaye MM. Spinal tuberculosis in HIV senegalese patients. Journal of the Neurological Sciences. 2017;381:424. doi: 10.1016/j.jns.2017.08.3410.

234. Marie Hoel I, Jorstad MD, Ruhwald M, Mustafa T, Dyrhol-Riise AM. IP-10 point-of-care tests for monitoring treatment efficacy in tuberculosis in a lowresource setting. European Respiratory Journal. 2017;50. doi: 10.1183/1393003.congress-2017.OA1950.

235. Mulu W, Abera B, Yimer M, Hailu T, Ayele H, Abate D. Rifampicin-resistance pattern of Mycobacterium tuberculosis and associated factors among presumptive tuberculosis patients referred to Debre Markos Referral Hospital, Ethiopia: a cross-sectional study. BMC research notes. 2017;10(1):8. Epub 2017/01/07. doi: 10.1186/s13104-016-2328-4. PubMed PMID: 28057041; PubMed Central PMCID: PMCPmc5217448.

236. Mulu W, Abera B, Yimer M, Hailu T, Ayele H, Abate D. Rifampicin-resistance pattern of Mycobacterium tuberculosis and associated factors among presumptive tuberculosis patients referred to Debre Markos Referral Hospital, Ethiopia: a cross-sectional study. BMC research notes. 2017;10(1):8. doi: 10.1186/s13104-016-2328-4.

237. Peghin M, Rodriguez-Pardo D, Sanchez-Montalva A, Pellise F, Rivas A, Tortola T, et al. The changing epidemiology of spinal tuberculosis: the influence of international immigration in Catalonia, 1993-2014. Epidemiology and infection. 2017;145(10):2152-60. Epub 2017/05/19. doi: 10.1017/s0950268817000863. PubMed PMID: 28516818.

238. Peghin M, Rodriguez-Pardo D, Sanchez-Montalva A, Pellisé F, Rivas A, Tortola T, et al. The changing epidemiology of spinal tuberculosis: The influence of international immigration in Catalonia, 1993-2014. Epidemiology and infection. 2017;145(10):2152-60. doi: 10.1017/S0950268817000863.

239. Peirse M, Houston A. Extrapulmonary tuberculosis. Medicine (United Kingdom). 2017;45(12):747-52. doi: 10.1016/j.mpmed.2017.09.008.

240. Pereira J, Anoop S, Pettah GJ. A Case of Chronic Inflammation of the Ankle Joint with Subtle Signs of Inflammation: A Rare Presentation of Tuberculosis Ankle. Journal of orthopaedic case reports. 2017;7(1):87-90. Epub 2017/06/21. doi: 10.13107/jocr.2250-0685.702. PubMed PMID: 28630849; PubMed Central PMCID: PMCPmc5458707.

241. Polepole P, Kabwe M, Kasonde M, Tembo J, Shibemba A, O'Grady J, et al. Performance of the Xpert MTB/RIF assay in the diagnosis of tuberculosis in formalin-fixed, paraffin-embedded tissues. International journal of mycobacteriology. 2017;6(1):87-93. Epub 2017/03/21. doi: 10.4103/2212-5531.201892. PubMed PMID: 28317811.

242. Polepole P, Kabwe M, Kasonde M, Tembo J, Shibemba A, O'Grady J, et al. Performance of the Xpert MTB/RIF assay in the diagnosis of tuberculosis in formalin-fixed, paraffin-embedded tissues. International journal of mycobacteriology. 2017;6(1):87-93. doi: 10.4103/2212-5531.201892.

243. Rufai SB, Singh A, Singh J, Kumar P, Sankar MM, Singh S. Diagnostic usefulness of Xpert MTB/RIF assay for detection of tuberculous meningitis using cerebrospinal fluid. The Journal of infection. 2017;75(2):125-31. Epub 2017/05/16. doi: 10.1016/j.jinf.2017.04.010. PubMed PMID: 28501491.

244. Rufai SB, Singh A, Singh J, Kumar P, Sankar MM, Singh S, et al. Diagnostic usefulness of Xpert MTB/RIF assay for detection of tuberculous meningitis using cerebrospinal fluid. Journal of Infection. 2017;75(2):125-31. doi: 10.1016/j.jinf.2017.04.010.

245. Shah PA, Coj M, Rohloff P. Delays in diagnosis and treatment of extrapulmonary tuberculosis in Guatemala. BMJ case reports. 2017;2017. doi: 10.1136/bcr-2017-220777.

246. Sharma SK, Chaubey J, Singh BK, Sharma R, Mittal A. Drug resistance pattern among extrapulmonary tuberculosis cases in a tertiary care center in India. American journal of respiratory and critical care medicine. 2017;195. doi: 10.1164/ajrccm-conference.2017.A61.

247. Sharma SK, Chaubey J, Singh BK, Sharma R, Mittal A, Sharma A. Drug resistance patterns among extra-pulmonary tuberculosis cases in a tertiary care centre in North India. The international journal of tuberculosis and lung disease : the official journal of the International Union against Tuberculosis and Lung Disease. 2017;21(10):1112-7. Epub 2017/09/16. doi: 10.5588/ijtld.16.0939. PubMed PMID: 28911354.

248. Sharma SK, Chaubey J, Singh BK, Sharma R, Mittal A, Sharma A. Drug resistance patterns among extra-pulmonary tuberculosis cases in a tertiary care centre in North India. International Journal of Tuberculosis and Lung Disease. 2017;21(10):1112-7. doi: 10.5588/ijtld.16.0939.

249. Sharma SK, Ryan H, Khaparde S, Sachdeva KS, Singh AD, Mohan A, et al. Index-TB guidelines: Guidelines on extrapulmonary tuberculosis for India. The Indian journal of medical research. 2017;145(4):448-63. Epub 2017/09/02. doi: 10.4103/ijmr.IJMR_1950_16. PubMed PMID: 28862176; PubMed Central PMCID: PMCPmc5663158.

250. Sharma SK, Ryan H, Khaparde S, Sachdeva KS, Singh AD, Mohan A, et al. Index-TB guidelines: Guidelines on extrapulmonary tuberculosis for India. Indian Journal of Medical Research. 2017;145(April):448-63. doi: 10.4103/ijmr.IJMR_1950_16.

251. Tadesse M, Abebe G, Bekele A, Bezabih M, de Rijk P, Meehan CJ, et al. The predominance of Ethiopian specific Mycobacterium tuberculosis families and minimal contribution of Mycobacterium bovis in tuberculous lymphadenitis patients in Southwest Ethiopia. Infection, genetics and evolution : journal of molecular epidemiology and evolutionary genetics in infectious diseases. 2017;55:251-9. Epub 2017/09/19. doi: 10.1016/j.meegid.2017.09.016. PubMed PMID: 28919549.

252. Tadesse M, Abebe G, Bekele A, Bezabih M, de Rijk P, Meehan CJ, et al. The predominance of Ethiopian specific Mycobacterium tuberculosis families and minimal contribution of Mycobacterium bovis in tuberculous lymphadenitis patients in Southwest Ethiopia. Infection, Genetics and Evolution. 2017;55:251-9. doi: 10.1016/j.meegid.2017.09.016.

253. Tang L, Feng S, Gao R, Han C, Sun X, Bao Y, et al. A Comparative Study on the Role of Xpert MTB/RIF in Testing Different Types of Spinal Tuberculosis Tissue Specimens. Genetic testing and molecular biomarkers. 2017;21(12):722-6. doi: 10.1089/gtmb.2017.0149.

254. Teng V, Chua Y, Lai E, Mukherjee S, Michaels J, Chia T, et al. Characteristics of HIV-TB infection in a middle TB burden country. International Journal of Antimicrobial Agents. 2017;50:S81-S2.

255. Tyagi S, Sharma N, Tyagi JS, Haldar S. Challenges in pleural tuberculosis diagnosis: existing reference standards and nucleic acid tests. Future microbiology. 2017;12:1201-18. Epub 2017/10/04. doi: 10.2217/fmb-2017-0028. PubMed PMID: 28972418.

256. Tyagi S, Sharma N, Tyagi JS, Haldar S. Challenges in pleural tuberculosis diagnosis: Existing reference standards and nucleic acid tests. Future microbiology. 2017;12(13):1201-18. doi: 10.2217/fmb-2017-0028.

257. Ullah I, Javaid A, Masud H, Ali M, Basit A, Ahmad W. Rapid detection of Mycobacterium tuberculosis and rifampicin resistance in extrapulmonary tuberculosis and sputum smear-negative pulmonary suspects using Xpert MTB/RIF. Journal of medical microbiology. 2017;66(4):412‐8. PubMed PMID: CN-01655405.

258. Ullah I, Javaid A, Masud H, Ali M, Basit A, Ahmad W, et al. Rapid detection of Mycobacterium tuberculosis and rifampicin resistance in extrapulmonary tuberculosis and sputum smear-negative pulmonary suspects using Xpert MTB/RIF. J Med Microbiol. 2017;66(4):412-8. Epub 2017/04/21. doi: 10.1099/jmm.0.000449. PubMed PMID: 28425873.

259. Ullah I, Javaid A, Masud H, Ali M, Basit A, Ahmad W, et al. Rapid detection of mycobacterium tuberculosis and rifampicin resistance in extrapulmonary tuberculosis and sputum smearnegative pulmonary suspects using Xpert MTB/RIF. Journal of Medical Microbiology. 2017;66(4):412-8. doi: 10.1099/jmm.0.000449.

260. Valencia S, Respeito D, Blanco S, Ribeiro RM, López-Varela E, Sequera VG, et al. Tuberculosis drug resistance in Southern Mozambique: Results of a population-level survey in the district of Manhiça. International Journal of Tuberculosis and Lung Disease. 2017;21(4):446-51. doi: 10.5588/ijtld.16.0694.

261. Van Hoving DJ, Meintjes G, Takwoingi Y, Griesel R, Maartens G, Ochodo EA. Abdominal ultrasound for diagnosing abdominal tuberculosis or disseminated tuberculosis with abdominal involvement in HIV‐positive adults. Cochrane Database of Systematic Reviews. 2017;(8). doi: 10.1002/14651858.CD012777. PubMed PMID: CD012777.

262. Wen H, Li P, Ma H, Lv G. Diagnostic accuracy of Xpert MTB/RIF assay for musculoskeletal tuberculosis: a meta-analysis. Infection and drug resistance. 2017;10:299-305. Epub 2017/10/14. doi: 10.2147/idr.s145843. PubMed PMID: 29026323; PubMed Central PMCID: PMCPmc5627758.

263. Wen H, Li P, Ma H, Lv G. Diagnostic accuracy of Xpert MTB/RIF assay for musculoskeletal tuberculosis: A meta-analysis. Infection and drug resistance. 2017;10:299-305. doi: 10.2147/IDR.S145843.

264. Yoon C, Semitala FC, Atuhumuza E, Katende J, Mwebe S, Asege L, et al. Point-of-care C-reactive protein-based tuberculosis screening for people living with HIV: a diagnostic accuracy study. The Lancet Infectious diseases. 2017;17(12):1285-92. Epub 2017/08/30. doi: 10.1016/s1473-3099(17)30488-7. PubMed PMID: 28847636; PubMed Central PMCID: PMCPmc5705273.

265. Zürcher K, Ballif M, Kiertiburanakul S, Yotebieng M, Grinsztejn B, Michael D, et al. Extrapulmonary TB at ART programs in lower-income countries: Diagnostics and outcomes. Topics in Antiviral Medicine. 2017;25(1):306s.

266. 金阳辉, 石仕元, 郑琦, 沈健, 应小樟, 汪翼凡. Xpert MTB/RIF在脊柱结核诊断及利福平耐药检测中的应用价值. 中国骨伤. 2017;30(9):787-91. doi: 10.3969/j.issn.1003-0034.2017.09.002.

267. 金阳辉, 石仕元, 郑琦, 沈健, 应小樟, 汪翼凡. Xpert MTB/RIF在脊柱结核诊断及利福平耐药检测中的应用价值. 中国骨伤. 2017;30(09):787-91.

268. 竺祖军, 夏强, 岳永宁, 张颖, 胡佳娜, 朱敏. Xpert MTB/RIF检测技术在骨关节结核诊断中的应用价值. 预防医学. 2017;29(03):322-4.

269. Aitzhanovna SL, Berikova E, Kurakbaevich KK, Anuarbekovich MT, Ergazievna AG, Arsen T, et al. Availability and economic feasibility (retrospective cohort study) of the implementation of rapid diagnostic methods for tuberculosis Xpert MTB / RIF in Kazakhstan. Research Journal of Pharmaceutical, Biological and Chemical Sciences. 2018;9(3):1478-85.

270. Ali A, Munir MA, Sarwar MI, Khaliq S, Munir MK, Rehman S. Tuberculous pleural effusions: Efficacy utilization and comparison of various diagnostic techniques. Pakistan Journal of Medical and Health Sciences. 2018;12(4):1461-4.

271. Ali A, Munir MA, Sarwar MI, Khaliq S, Munir MK, Rehman S. Tuberculous pleural effusions: Efficacy utilization and comparison of various diagnostic techniques. Pakistan Journal of Medical and Health Sciences. 2018;12(4):1570-3.

272. Anam MS, Dadiyanto DW, Sidhartani M. Characteristics of tuberculosis in children in Kariadi Hospital semarang Indonesia. Pediatric Pulmonology. 2018;53:S116-S7. doi: 10.1002/ppul.24034.

273. Andarini I. Clinical features of tuberculosis in pediatrics. Pediatric Pulmonology. 2018;53:S116. doi: 10.1002/ppul.24034.

274. Bankar S, Set R, Sharma D, Shah D, Shastri J. Diagnostic accuracy of Xpert MTB/RIF assay in extrapulmonary tuberculosis. Indian journal of medical microbiology. 2018;36(3):357-63. Epub 2018/11/16. doi: 10.4103/ijmm.IJMM_18_173. PubMed PMID: 30429387.

275. Bankar S, Set R, Sharma D, Shah D, Shastri J. Diagnostic accuracy of Xpert MTB/RIF assay in extrapulmonary tuberculosis. Indian journal of medical microbiology. 2018;36(3):357-63. doi: 10.4103/ijmm.IJMM_18_173.

276. Ben Saad S, Kallel N, Gharsalli H, Kwas H, El Gharbi L, Ghedira H, et al. Cold abscess in the immunocompetent subject. La Tunisie medicale. 2018;96(5):302-6. Epub 2018/11/16. PubMed PMID: 30430505.

277. Ben Saad S, Kallel N, Gharsalli H, Kwas H, El Gharbi L, Ghedira H, et al. Cold abscess in the immunocompetent subject. Tunisie Medicale. 2018;96(5):302-6.

278. Broderick C, Hopkins S, Mack DJF, Aston W, Pollock R, Skinner JA, et al. Delays in the diagnosis and treatment of bone and joint tuberculosis in the United Kingdom. The bone & joint journal. 2018;100-b(1):119-24. Epub 2018/01/07. doi: 10.1302/0301-620x.100b1.bjj-2017-0357.r1. PubMed PMID: 29305460.

279. Broderick C, Hopkins S, Mack DJF, Aston W, Pollock R, Skinner JA, et al. Delays in the diagnosis and treatment of bone and joint tuberculosis in the United Kingdom. Bone and Joint Journal. 2018;100B(1):119-24. doi: 10.1302/0301-620X.100B1.BJJ-2017-0357.R1.

280. Budgell EP, Evans D, Leuner R, Long L, Rosen S. The costs and outcomes of paediatric tuberculosis treatment at primary healthcare clinics in Johannesburg, South Africa. South African Medical Journal. 2018;108(5):423-31. doi: 10.7196/SAMJ.2018.v108i5.12802.

281. Christopher DJ, Dinakaran S, Gupta R, James P, Isaac B, Thangakunam B. Thoracoscopic pleural biopsy improves yield of Xpert MTB/RIF for diagnosis of pleural tuberculosis. Respirology (Carlton, Vic). 2018;23(7):714-7. doi: 10.1111/resp.13275.

282. Colon FA, Gabayoyo EL, Gabayoyo M. Extra pulmonary tuberculosis in HIV patients: Profile & outcome of patients at Western Visayasmedical Center PPMD dots unit Philippines January 2015 to June 2018. Respirology (Carlton, Vic). 2018;23:205-6. doi: 10.1111/resp.13420_319.

283. Davis R, Higgens C, Cosgrove C, Shur J, Arkell P. Tuberculous arthritis: negative Xpert MTB/RIF assay does not rule out infection! BMJ case reports. 2018;2018. Epub 2018/07/01. doi: 10.1136/bcr-2018-224288. PubMed PMID: 29959172.

284. Davis R, Higgens C, Cosgrove C, Shur J, Arkell P. Tuberculous arthritis: Negative Xpert MTB/RIF assay does not rule out infection! BMJ case reports. 2018;2018. doi: 10.1136/bcr-2018-224288.

285. Elzein F, Elzein A, Mohammed N, Alswailem R. Miliary tuberculosis mimicking systemic lupus erythematosus flare. Respiratory Medicine Case Reports. 2018;25:216-9. doi: 10.1016/j.rmcr.2018.09.005.

286. Fan L, Li D, Zhang S, Yao L, Hao X, Gu J, et al. Parallel Tests Using Culture, Xpert MTB/RIF, and SAT-TB in Sputum Plus Bronchial Alveolar Lavage Fluid Significantly Increase Diagnostic Performance of Smear-Negative Pulmonary Tuberculosis. Frontiers in microbiology. 2018;9:1107. Epub 2018/07/06. doi: 10.3389/fmicb.2018.01107. PubMed PMID: 29973917; PubMed Central PMCID: PMCPmc6020777.

287. Galloway KM, Parker R. Could an increase in vigilance for spinal tuberculosis at primary health care level, enable earlier diagnosis at district level in a tuberculosis endemic country? African journal of primary health care & family medicine. 2018;10(1):e1-e9. Epub 2018/06/27. doi: 10.4102/phcfm.v10i1.1666. PubMed PMID: 29943617; PubMed Central PMCID: PMCPmc6018652.

288. Galloway KM, Parker R. Could an increase in vigilance for spinal tuberculosis at primary health care level, enable earlier diagnosis at district level in a tuberculosis endemic country? African journal of primary health care & family medicine. 2018;10(1):e1-e9. doi: 10.4102/phcfm.v10i1.1666.

289. Garcia R, Yamshon S. Disseminated tuberculosis masquerading as a neuroendocrine tumour. BMJ case reports. 2018;2018. doi: 10.1136/bcr-2018-225591.

290. García-Basteiro AL, DiNardo A, Saavedra B, Silva DR, Palmero D, Gegia M, et al. Point of care diagnostics for tuberculosis. Revista Portuguesa de Pneumologia (English Edition). 2018;24(2):73-85. doi: 10.1016/j.rppnen.2017.12.002.

291. Gati S, Chetty R, Wilson D, Achkar JM. Utilization and clinical value of diagnostic modalities for tuberculosis in a high HIV prevalence setting. American Journal of Tropical Medicine and Hygiene. 2018;99(2):317-22. doi: 10.4269/ajtmh.17-0965.

292. Gautam H, Agrawal SK, Verma SK, Singh UB. Cervical tuberculous lymphadenitis: Clinical profile and diagnostic modalities. International journal of mycobacteriology. 2018;7(3):212-6. Epub 2018/09/11. doi: 10.4103/ijmy.ijmy_99_18. PubMed PMID: 30198498.

293. Gautam H, Agrawal SK, Verma SK, Singh UB. Cervical tuberculous lymphadenitis: Clinical profile and diagnostic modalities. International journal of mycobacteriology. 2018;7(3):212-6. doi: 10.4103/ijmy.ijmy_99_18.

294. Gibson J, Coucher J, Coulter C, Eather G. Pleuropulmonary tuberculosis with spinal lesions due to metastatic malignancy differentiated definitively on imaging. BMJ case reports. 2018;11(1). doi: 10.1136/bcr-2018-226160.

295. Gounden S, Perumal R, Magula NP. Extrapulmonary tuberculosis in the setting of HIV hyperendemicity at a tertiary hospital in Durban, South Africa. Southern African Journal of Infectious Diseases. 2018;33(3):57-64. doi: 10.1080/23120053.2017.1403207.

296. Gupta M, Purohit G, Vyas S. Prevalence of positivity of CBNAAT(cartridge based nucleic acid amplification test) in extra-pulmonary tuberculosis. European Respiratory Journal. 2018;52. doi: 10.1183/13993003.congress-2018.PA2756.

297. Gupta-Wright A, Corbett EL, van Oosterhout JJ, Wilson D, Grint D, Alufandika-Moyo M, et al. Rapid urine-based screening for tuberculosis in HIV-positive patients admitted to hospital in Africa (STAMP): a pragmatic, multicentre, parallel-group, double-blind, randomised controlled trial. Lancet (London, England). 2018;392(10144):292-301. Epub 2018/07/24. doi: 10.1016/s0140-6736(18)31267-4. PubMed PMID: 30032978; PubMed Central PMCID: PMCPmc6078909.

298. Haraka F, Nathavitharana RR, Schumacher SG, Kakolwa M, Denkinger CM, Gagneux S, et al. Impact of diagnostic test Xpert MTB/RIF® on health outcomes for tuberculosis. Cochrane Database of Systematic Reviews. 2018;(2). doi: 10.1002/14651858.CD012972. PubMed PMID: CD012972.

299. Haraka F, Nathavitharana RR, Schumacher SG, Kakolwa M, Denkinger CM, Gagneux S, et al. Impact of diagnostic test Xpert MTB/RIF® on health outcomes for tuberculosis. Cochrane Database of Systematic Reviews. 2018;2018(2). doi: 10.1002/14651858.CD012972.

300. Jain AK, Jaggi KR, Bhayana H, Saha R. Drug-resistant Spinal Tuberculosis. Indian journal of orthopaedics. 2018;52(2):100-7. Epub 2018/03/27. doi: 10.4103/ortho.IJOrtho_306_17. PubMed PMID: 29576636; PubMed Central PMCID: PMCPmc5858202.

301. Jorstad MD, Marijani M, Dyrhol-Riise AM, Sviland L, Mustafa T. MPT64 antigen detection test improves routine diagnosis of extrapulmonary tuberculosis in a low-resource setting: A study from the tertiary care hospital in Zanzibar. PloS one. 2018;13(5):e0196723. Epub 2018/05/10. doi: 10.1371/journal.pone.0196723. PubMed PMID: 29742144; PubMed Central PMCID: PMCPmc5942825.

302. Jørstad MD, Marijani M, Dyrhol-Riise AM, Sviland L, Mustafa T. MPT64 antigen detection test improves routine diagnosis of extrapulmonary tuberculosis in a low-resource setting: A study from the tertiary care hospital in Zanzibar. PloS one. 2018;13(5). doi: 10.1371/journal.pone.0196723.

303. Kanade S, Nataraj G, Mehta P. Improved case detection using Xpert Mycobacterium tuberculosis/rifampicin assay in skeletal tuberculosis. Indian journal of medical microbiology. 2018;36(4):590-3. doi: 10.4103/ijmm.IJMM_19_10. PubMed Central PMCID: PMChttp://www.ijmm.org/article.asp?issn=0255-0857;year=2018;volume=36;issue=4;spage=590;epage=593;aulast=Kanade.

304. Khan AS, Ali S, Khan MT, Ahmed S, Khattak Y, Abduljabbar, et al. Comparison of GeneXpert MTB/RIF assay and LED-FM microscopy for the diagnosis of extra pulmonary tuberculosis in Khyber Pakhtunkhwa, Pakistan. Brazilian journal of microbiology : [publication of the Brazilian Society for Microbiology]. 2018;49(4):909-13. doi: 10.1016/j.bjm.2018.02.011.

305. Khose S, Kharaje J. Poncet's disease-Elbow joint tuberculosis presentng with seropositve polyarthrits: A rare entty. Indian Journal of Rheumatology. 2018;13(6):S166.

306. Kohli M, Schiller I, Dendukuri N, Dheda K, Denkinger CM, Schumacher SG, et al. Xpert® MTB/RIF assay for extrapulmonary tuberculosis and rifampicin resistance. Cochrane Database of Systematic Reviews. 2018;(8). doi: 10.1002/14651858.CD012768.pub2. PubMed PMID: CD012768.

307. Kohli M, Schiller I, Dendukuri N, Dheda K, Denkinger CM, Schumacher SG, et al. Xpert((R)) MTB/RIF assay for extrapulmonary tuberculosis and rifampicin resistance. The Cochrane database of systematic reviews. 2018;8:Cd012768. Epub 2018/08/28. doi: 10.1002/14651858.CD012768.pub2. PubMed PMID: 30148542.

308. Kohli M, Schiller I, Dendukuri N, Dheda K, Denkinger CM, Schumacher SG, et al. Xpert® MTB/RIF assay for extrapulmonary tuberculosis and rifampicin resistance. Cochrane Database of Systematic Reviews. 2018;2018(8). doi: 10.1002/14651858.CD012768.pub2.

309. Li Y, Jia W, Lei G, Zhao D, Wang G, Qin S. Diagnostic efficiency of Xpert MTB/RIF assay for osteoarticular tuberculosis in patients with inflammatory arthritis in China. PloS one. 2018;13(6). doi: 10.1371/journal.pone.0198600.

310. Liu C, Lyon CJ, Bu Y, Deng Z, Walters E, Li Y, et al. Clinical evaluation of a blood assay to diagnose paucibacillary tuberculosis via bacterial antigens. Clinical Chemistry. 2018;64(5):791-800. doi: 10.1373/clinchem.2017.273698.

311. Maruthai K, Kalaiarasan E, Joseph NM, Parija SC, Mahadevan S. Assessment of global DNA methylation in children with tuberculosis disease. International journal of mycobacteriology. 2018;7(4):338-42. doi: 10.4103/ijmy.ijmy_107_18.

312. Metaferia Y, Seid A, Fenta GM, Gebretsadik D. Assessment of Extrapulmonary Tuberculosis Using Gene Xpert MTB/RIF Assay and Fluorescent Microscopy and Its Risk Factors at Dessie Referral Hospital, Northeast Ethiopia. BioMed research international. 2018;2018:8207098. Epub 2018/08/31. doi: 10.1155/2018/8207098. PubMed PMID: 30159328; PubMed Central PMCID: PMCPmc6106971.

313. Metaferia Y, Seid A, Fenta GM, Gebretsadik D. Assessment of Extrapulmonary Tuberculosis Using Gene Xpert MTB/RIF Assay and Fluorescent Microscopy and Its Risk Factors at Dessie Referral Hospital, Northeast Ethiopia. BioMed research international. 2018;2018. doi: 10.1155/2018/8207098.

314. Mugomeri E, Bekele BS, Maibvise C, Tarirai C. Trends in diagnostic techniques and factors associated with tuberculosis treatment outcomes in Lesotho, 2010–2015. Southern African Journal of Infectious Diseases. 2018;33(1):18-23. doi: 10.1080/23120053.2017.1376545.

315. Oliwa JN, Maina J, Ayieko P, Gathara D, Kathure IA, Masini E, et al. Variability in distribution and use of tuberculosis diagnostic tests in Kenya: A cross-sectional survey. BMC infectious diseases. 2018;18(1). doi: 10.1186/s12879-018-3237-z.

316. Perez-Risco D, Rodriguez-Temporal D, Valledor-Sanchez I, Alcaidea F. Evaluation of the Xpert MTB/RIF Ultra Assay for Direct Detection of Mycobacterium tuberculosis Complex in Smear-Negative Extrapulmonary Samples. Journal of Clinical Microbiology. 2018;56(9). doi: 10.1128/JCM.00659-18.

317. Prakash AK, Datta B, Tripathy JP, Kumar N, Chatterjee P, Jaiswal A. The clinical utility of cycle of threshold value of GeneXpert MTB/RIF (CBNAAT) and its diagnostic accuracy in pulmonary and extra-pulmonary samples at a tertiary care center in India. Indian Journal of Tuberculosis. 2018;65(4):296-302. doi: 10.1016/j.ijtb.2018.05.021.

318. Raizada N, Khaparde SD, Rao R, Kalra A, Sarin S, Salhotra VS, et al. Upfront Xpert MTB/RIF testing on various specimen types for presumptive infant TB cases for early and appropriate treatment initiation. PloS one. 2018;13(8). doi: 10.1371/journal.pone.0202085.

319. Raizada N, Khaparde SD, Salhotra VS, Rao R, Kalra A, Swaminathan S, et al. Accelerating access to quality TB care for pediatric TB cases through better diagnostic strategy in four major cities of India. PloS one. 2018;13(2). doi: 10.1371/journal.pone.0193194.

320. Raizada N, Khaparde SD, Swaminathan S, Sarin S, Salhotra VS, Kalra A, et al. Catalysing progressive uptake of newer diagnostics by health care providers through outreach and education in four major cities of India. PloS one. 2018;13(3). doi: 10.1371/journal.pone.0193341.

321. Rajasekaran S, Soundararajan DCR, Shetty AP, Kanna RM. Spinal Tuberculosis: Current Concepts. Global spine journal. 2018;8(4 Suppl):96s-108s. Epub 2018/12/24. doi: 10.1177/2192568218769053. PubMed PMID: 30574444; PubMed Central PMCID: PMCPmc6295815.

322. Rajasekaran S, Soundararajan DCR, Shetty AP, Kanna RM. Spinal Tuberculosis: Current Concepts. Global spine journal. 2018;8(4_suppl):96S-108S. doi: 10.1177/2192568218769053.

323. Rakotoarivelo R, Ambrosioni J, Rasolofo V, Raberahona M, Rakotosamimanana N, Andrianasolo R, et al. Evaluation of the Xpert MTB/RIF assay for the diagnosis of smear-negative pulmonary and extrapulmonary tuberculosis in Madagascar. International journal of infectious diseases : IJID : official publication of the International Society for Infectious Diseases. 2018;69:20-5. Epub 2018/02/07. doi: 10.1016/j.ijid.2018.01.017. PubMed PMID: 29408360.

324. Rakotoarivelo R, Ambrosioni J, Rasolofo V, Raberahona M, Rakotosamimanana N, Andrianasolo R, et al. Evaluation of the Xpert MTB/RIF assay for the diagnosis of smear-negative pulmonary and extrapulmonary tuberculosis in Madagascar. International Journal of Infectious Diseases. 2018;69:20-5. doi: 10.1016/j.ijid.2018.01.017.

325. Sachdeva K, Shrivastava T. CBNAAT: A Boon for Early Diagnosis of Tuberculosis-Head and Neck. Indian journal of otolaryngology and head and neck surgery : official publication of the Association of Otolaryngologists of India. 2018;70(4):572-7. Epub 2018/11/23. doi: 10.1007/s12070-018-1364-x. PubMed PMID: 30464918; PubMed Central PMCID: PMCPmc6224834.

326. Sanke S, Chander R, Dalal K, Agarwal S. Metastatic tubercular gummas and splenic tuberculoma secondary to tubercular lymphadenitis in an immunocompetent female. International Journal of Dermatology. 2018;57(10):1229-32. doi: 10.1111/ijd.14085.

327. Shakeel K, Iram S, Akhtar M, Hussain S, Maryam H, Anwar A. Diagnostic validation of rapid molecular detection of Mycobacterium tuberculosis in pus samples by GeneXpert. JPMA The Journal of the Pakistan Medical Association. 2018;68(1):33-7. Epub 2018/01/27. PubMed PMID: 29371714.

328. Shakeel K, Iram S, Akhtar M, Hussain S, Maryam H, Anwar A. Diagnostic validation of rapid molecular detection of Mycobacterium tuberculosis in pus samples by GeneXpert®. Journal of the Pakistan Medical Association. 2018;68(1):33-7.

329. Shetty A, Kanna RM, Maheswaran A, Bhari P, Rajasekaran S. Whole spine magnetic resonance imaging findings are highly sensitive in the diagnosis of spinal tuberculosis. Global spine journal. 2018;8(1):287S. doi: 10.1177/2192568218771072.

330. Solanki A, Basu S, Biswas A, Banta A. Accuracy of gene xpert as diagnostic tool in spinal tuberculosis. Global spine journal. 2018;8(1):51S. doi: 10.1177/2192568218771030.

331. Tadesse M, Abebe G, Bekele A, Bezabih M, Yilma D, Apers L, et al. Xpert MTB/RIF assay for the diagnosis of extrapulmonary tuberculosis: a diagnostic evaluation study. Clinical microbiology and infection : the official publication of the European Society of Clinical Microbiology and Infectious Diseases. 2018. Epub 2018/12/26. doi: 10.1016/j.cmi.2018.12.018. PubMed PMID: 30583052.

332. Tang Y, Yin L, Tang S, Zhang H, Lan J. Application of molecular, microbiological, and immunological tests for the diagnosis of bone and joint tuberculosis. Journal of clinical laboratory analysis. 2018;32(2). doi: 10.1002/jcla.22260.

333. Tiresse N, Abid A. [Spinal cord compression in a patient with tuberculous spondylodiscitis]. The Pan African medical journal. 2018;31:101. Epub 2019/05/01. doi: 10.11604/pamj.2018.31.101.17054. PubMed PMID: 31037162; PubMed Central PMCID: PMCPmc6462379.

334. Tiresse N, Abid A. Spinal cord compression in a patient with tuberculous spondylodiscitis. Pan African Medical Journal. 2018;31. doi: 10.11604/pamj.2018.31.101.17054.

335. Tseng S, Dick CR, Seroy J. Miliary tuberculosis complicated by meningitis and intracranial tuberculomas in pregnancy. American journal of respiratory and critical care medicine. 2018;197(MeetingAbstracts).

336. Ukunda FUN. Atypical infections of the spine in the context of HIV and TB co-infection. Global spine journal. 2018;8(1):63S. doi: 10.1177/2192568218771030.

337. Usman S. Impact of geneXpert MTB/RIF for rapid tuberculosis diagnosis and rifampicin resistance detection among PLWHIV in South-Western Nigeria. Journal of the International AIDS Society. 2018;21:103. doi: 10.1002/jia2.25187.

338. Walzl G, McNerney R, du Plessis N, Bates M, McHugh TD, Chegou NN, et al. Tuberculosis: advances and challenges in development of new diagnostics and biomarkers. The Lancet Infectious diseases. 2018;18(7):e199-e210. Epub 2018/03/28. doi: 10.1016/s1473-3099(18)30111-7. PubMed PMID: 29580818.

339. Walzl G, McNerney R, du Plessis N, Bates M, McHugh TD, Chegou NN, et al. Tuberculosis: advances and challenges in development of new diagnostics and biomarkers. The Lancet Infectious Diseases. 2018;18(7):e199-e210. doi: 10.1016/S1473-3099(18)30111-7.

340. Wang G, Dong W, Lan T, Fan J, Tang K, Li Y, et al. Diagnostic accuracy evaluation of the conventional and molecular tests for Spinal Tuberculosis in a cohort, head-to-head study. Emerging Microbes and Infections. 2018;7(1). doi: 10.1038/s41426-018-0114-1.

341. 侯婷婷, 侯惺, 李远, 李钿, 索海燕, 周正. GeneXpert MTB/RIF在检测脊柱结核患者中的诊断价值. 检验医学与临床. 2018;15(21):3268-70.

342. 贾晨光, 高建国, 姚晓伟, 王连波, 姚黎明, 李雯. Xpert MTB/RIF技术在疑似脊柱结核早期诊断及利福平耐药检测中的价值研究. 河北医科大学学报. 2018;39(09):1031-3+48.

343. 刘红伟, 李晓非, 黄山, 梁桂亮. GeneXpert MTB/RIF检测技术在关节结核中的诊断价值. 中国医刊. 2018;53(06):688-91.

344. Arockiaraj J, Karthik R, Michael JS, Amritanand R, David KS, Krishnan V, et al. 'Need of the Hour': Early Diagnosis and Management of Multidrug Resistant Tuberculosis of the Spine: An Analysis of 30 Patients from a "High Multidrug Resistant Tuberculosis Burden" Country. Asian spine journal. 2019;13(2):265-71. Epub 2019/01/24. doi: 10.31616/asj.2018.0073. PubMed PMID: 30669824; PubMed Central PMCID: PMCPmc6454281.

345. Arockiaraj J, Robert M, Rose W, Amritanand R, David KS, Krishnan V. Early Detection and Analysis of Children with Multidrug-Resistant Tuberculosis of the Spine. Asian spine journal. 2019;13(1):77-85. Epub 2018/10/18. doi: 10.31616/asj.2017.0217. PubMed PMID: 30326699; PubMed Central PMCID: PMCPmc6365795.

346. Di Tanna GL, Khaki AR, Theron G, McCarthy K, Cox H, Mupfumi L, et al. Effect of Xpert MTB/RIF on clinical outcomes in routine care settings: individual patient data meta-analysis. The Lancet Global health. 2019;7(2):e191-e9. Epub 2019/01/27. doi: 10.1016/s2214-109x(18)30458-3. PubMed PMID: 30683238; PubMed Central PMCID: PMCPmc6366854.

347. Di Tanna GL, Raza Khaki A, Theron G, McCarthy K, Cox H, Mupfumi L, et al. Effect of Xpert MTB/RIF on clinical outcomes in routine care settings: individual patient data meta-analysis. The Lancet Global Health. 2019;7(2):e191-e9. doi: 10.1016/S2214-109X(18)30458-3.

348. Hoel IM, Jorstad MD, Marijani M, Ruhwald M, Mustafa T, Dyrhol-Riise AM. IP-10 dried blood spots assay monitoring treatment efficacy in extrapulmonary tuberculosis in a low-resource setting. Scientific reports. 2019;9(1):3871. Epub 2019/03/09. doi: 10.1038/s41598-019-40458-0. PubMed PMID: 30846768; PubMed Central PMCID: PMCPmc6405853.

349. Hoel IM, Jørstad MD, Marijani M, Ruhwald M, Mustafa T, Dyrhol-Riise AM. IP-10 dried blood spots assay monitoring treatment efficacy in extrapulmonary tuberculosis in a low-resource setting. Scientific reports. 2019;9(1):3871. doi: 10.1038/s41598-019-40458-0.

350. Khan SI, Ahmed N, Rahman A, Al Mahmud A, Islam R, Ahsan MK. Tuberculous spondylitis involving sacrum-a rare location and presentation of Pott’s disease: Case report and review of the literature. Bangladesh Journal of Medical Science. 2019;18(2):440-5. doi: 10.3329/bjms.v18i2.40724.

351. Kumari P, Lavania S, Tyagi S, Dhiman A, Rath D, Anthwal D, et al. A novel aptamer-based test for the rapid and accurate diagnosis of pleural tuberculosis. Analytical biochemistry. 2019;564-565:80-7. Epub 2018/10/24. doi: 10.1016/j.ab.2018.10.019. PubMed PMID: 30352198.

352. Kumari P, Lavania S, Tyagi S, Dhiman A, Rath D, Anthwal D, et al. A novel aptamer-based test for the rapid and accurate diagnosis of pleural tuberculosis. Analytical biochemistry. 2019;564-565:80-7. doi: 10.1016/j.ab.2018.10.019.

353. Mbuh TP, Ane-Anyangwe I, Adeline W, Thumamo Pokam BD, Meriki HD, Mbacham WF. Bacteriologically confirmed extra pulmonary tuberculosis and treatment outcome of patients consulted and treated under program conditions in the littoral region of Cameroon. BMC pulmonary medicine. 2019;19(1):17. Epub 2019/01/19. doi: 10.1186/s12890-018-0770-x. PubMed PMID: 30654769; PubMed Central PMCID: PMCPmc6337766.

354. Mbuh TP, Ane-Anyangwe I, Adeline W, Thumamo Pokam BD, Meriki HD, Mbacham WF. Bacteriologically confirmed extra pulmonary tuberculosis and treatment outcome of patients consulted and treated under program conditions in the littoral region of Cameroon 11 Medical and Health Sciences 1103 Clinical Sciences. BMC pulmonary medicine. 2019;19(1). doi: 10.1186/s12890-018-0770-x.

355. Nataprawira HM, Handisurya IWA, Adrian N. INCREASING TREND OF TB AMONG ADOLESCENTS IN A HIGH-BURDEN SETTING. Chest. 2019;155(4):239A. doi: 10.1016/j.chest.2019.02.228.

356. Ndege R, Weisser M, Elzi L, Diggelmann F, Bani F, Gingo W, et al. Sonography to Rule Out Tuberculosis in Sub-Saharan Africa: A Prospective Observational Study. Open forum infectious diseases. 2019;6(4):ofz154. Epub 2019/05/02. doi: 10.1093/ofid/ofz154. PubMed PMID: 31041350; PubMed Central PMCID: PMCPmc6483805.

357. Negi SS, Singh P, Chandrakar S, Gaikwad U, Das P, Bhargava A, et al. Diagnostic evaluation of multiplex real time PCR, genexpert MTB/RIF assay and conventional methods in extrapulmonary tuberculosis. Journal of Clinical and Diagnostic Research. 2019;13(1):DC12-DC6. doi: 10.7860/JCDR/2019/37569.12485.

358. Oliosi JGN, Reis-Santos B, Locatelli RL, Sales CMM, da Silva Filho WG, da Silva KC, et al. Effect of the Bolsa Familia Programme on the outcome of tuberculosis treatment: a prospective cohort study. The Lancet Global Health. 2019;7(2):e219-e26. doi: 10.1016/S2214-109X(18)30478-9.

359. Opota O, Mazza-Stalder J, Greub G, Jaton K. The rapid molecular test Xpert MTB/RIF ultra: towards improved tuberculosis diagnosis and rifampicin resistance detection. Clinical microbiology and infection : the official publication of the European Society of Clinical Microbiology and Infectious Diseases. 2019. Epub 2019/04/01. doi: 10.1016/j.cmi.2019.03.021. PubMed PMID: 30928564.

360. Opota O, Mazza-Stalder J, Greub G, Jaton K. The rapid molecular test Xpert MTB/RIF ultra: towards improved tuberculosis diagnosis and rifampicin resistance detection. Clinical Microbiology and Infection. 2019. doi: 10.1016/j.cmi.2019.03.021.

361. Piersimoni C, Gherardi G, Gracciotti N, Pocognoli A. Comparative evaluation of Xpert MTB/RIF and the new Xpert MTB/RIF ultra with respiratory and extra-pulmonary specimens for tuberculosis case detection in a low incidence setting. Journal of Clinical Tuberculosis and Other Mycobacterial Diseases. 2019;15. doi: 10.1016/j.jctube.2019.100094.

362. Shakoor S, Mir F, Hasan R. Common alternative diagnoses among a pediatric hospital-based cohort evaluated for tuberculosis in Karachi, Pakistan: The need for facilitated referral in tuberculosis clinics. International journal of mycobacteriology. 2019;8(1):42-7. doi: 10.4103/ijmy.ijmy_8_19.

363. Sharma S, Sheoran A, Gupta KB, Yadav A, Varma-Basil M, Sreenivas V, et al. Quantitative detection of a cocktail of mycobacterial MPT64 and PstS1 in tuberculosis patients by real-time immuno-PCR. Future microbiology. 2019;14(3):223-34. doi: 10.2217/fmb-2018-0284.

364. Singh P, Saket VK, Kachhi R. Diagnosis of TB: From conventional to modern molecular protocols. Frontiers in Bioscience - Elite. 2019;11(1):38-60. doi: 10.2741/E845.

365. Tadesse M, Abebe G, Bekele A, Bezabih M, Yilma D, Apers L, et al. Xpert MTB/RIF assay for the diagnosis of extrapulmonary tuberculosis: a diagnostic evaluation study. Clinical Microbiology and Infection. 2019. doi: 10.1016/j.cmi.2018.12.018.

366. Vijayageetha M, Kumar AM, Ramakrishnan J, Sarkar S, Papa D, Mehta K, et al. Tuberculosis screening among pregnant women attending a tertiary care hospital in Puducherry, South India: is it worth the effort? Global health action. 2019;12(1):1564488. Epub 2019/02/27. doi: 10.1080/16549716.2018.1564488. PubMed PMID: 30806593; PubMed Central PMCID: PMCPmc6394292.

367. Wu X, Tan G, Gao R, Yao L, Bi D, Guo Y, et al. Assessment of the Xpert MTB/RIF Ultra assay on rapid diagnosis of extrapulmonary tuberculosis. International journal of infectious diseases : IJID : official publication of the International Society for Infectious Diseases. 2019;81:91-6. Epub 2019/02/11. doi: 10.1016/j.ijid.2019.01.050. PubMed PMID: 30738907.

368. Wu X, Tan G, Gao R, Yao L, Bi D, Guo Y, et al. Assessment of the Xpert MTB/RIF Ultra assay on rapid diagnosis of extrapulmonary tuberculosis. International Journal of Infectious Diseases. 2019;81:91-6. doi: 10.1016/j.ijid.2019.01.050.

369. Yoon C, Semitala FC, Asege L, Katende J, Mwebe S, Andama AO, et al. Yield and Efficiency of Novel Intensified Tuberculosis Case-Finding Algorithms for People Living with HIV. American journal of respiratory and critical care medicine. 2019;199(5):643-50. Epub 2018/09/08. doi: 10.1164/rccm.201803-0490OC. PubMed PMID: 30192649; PubMed Central PMCID: PMCPmc6396864.

370. 饶敬澄, 蔡玉强, 曹广如, 杨志花. Xpert MTB/RIF在脊柱结核快速诊断及利福平耐药性 检测的应用分析. 中国伤残医学. 2019;27(7):32-3. doi: 10.13214/j.cnki.cjotadm.2019.07.019.

371. Ndege R, Weisser M, Elzi L, Diggelmann F, Bani F, Gingo W, et al. Sonography to Rule Out Tuberculosis in Sub-Saharan Africa: A Prospective Observational Study. Open forum infectious diseases. 2019;6(4):ofz154. Epub 2019/05/02. doi: 10.1093/ofid/ofz154. PubMed PMID: 31041350; PubMed Central PMCID: PMCPmc6483805.

372. Negi SS, Singh P, Chandrakar S, Gaikwad U, Das P, Bhargava A, et al. Diagnostic evaluation of multiplex real time PCR, genexpert MTB/RIF assay and conventional methods in extrapulmonary tuberculosis. Journal of Clinical and Diagnostic Research. 2019;13(1):DC12-DC6. doi: 10.7860/JCDR/2019/37569.12485.

373. Valencia S, Respeito D, Blanco S, Ribeiro RM, López-Varela E, Sequera VG, et al. Tuberculosis drug resistance in Southern Mozambique: Results of a population-level survey in the district of Manhiça. International Journal of Tuberculosis and Lung Disease. 2017;21(4):446-51. doi: 10.5588/ijtld.16.0694.

374. Van Hoving DJ, Meintjes G, Takwoingi Y, Griesel R, Maartens G, Ochodo EA. Abdominal ultrasound for diagnosing abdominal tuberculosis or disseminated tuberculosis with abdominal involvement in HIV‐positive adults. Cochrane Database of Systematic Reviews. 2017;(8). doi: 10.1002/14651858.CD012777.
