# Supplementary material for: Diagnostic accuracy of the Xpert MTB/RIF assay for bone and joint tuberculosis: A meta-analysis
Source: PLoS One. 2019 Aug 22;14(8):e0221427. doi: 10.1371/journal.pone.0221427 (PMC6705841; doi:10.1371/journal.pone.0221427)
Supplement: S1 Supporting Information. Data — (ZIP) [file pone.0221427.s001.zip › S1 Supporting Information/Search strategy.docx]

The catalogues of search terms in PubMed (MeSH) and EMBASE (Emtree) are different, therefore, the theme words of the same disease are different in the two database. The first step in retrieval is to determine the theme words, the theme words of osteoarticular tuberculosis and spinal tuberculosis are "Tuberculosis, Osteoarticular"[Mesh], "Tuberculosis, Spinal"[Mesh] and 'tuberculous arthritis'/exp, 'tuberculous spondylitis'/exp in PubMed (MeSH) and EMBASE (Emtree), respectively.

Pubmed and Cochrane:

#1 "Tuberculosis, Osteoarticular"[Mesh]

#2 “Osteoarticular Tuberculoses” OR “Osteoarticular Tuberculosis” OR “Tuberculoses, Osteoarticular” OR “Bone Tuberculosis” OR “Bone Tuberculoses” OR “Tuberculoses, Bone” OR “Tuberculosis, Bone” OR “Joint Tuberculosis” OR “Joint Tuberculoses” OR “Tuberculoses, Joint” OR “Tuberculosis, Joint” OR “tuberculous arthritis” OR “musculoskeletal tuberculosis”

#3 "Tuberculosis, Spinal"[Mesh]

#4 “Spinal Tuberculoses” OR “Spinal Tuberculosis” OR “Tuberculoses, Spinal” OR “Pott Disease” OR “Disease, Pott” OR “Pott's Disease” OR “Disease, Pott's” OR “Potts Disease” OR “Pott's Paraplegia” OR “tuberculous spondylitis”

#5 “Extrapulmonary tuberculosis” OR “Extra pulmonary tuberculosis”

#6 #1 OR #2 OR #3 OR #4 OR #5

#7 Xpert OR genexpert

#8 #6 And #7

Embase

#1 'tuberculous arthritis'/exp

#2 ‘Osteoarticular Tuberculoses’ OR ‘Osteoarticular Tuberculosis’ OR ‘Tuberculoses, Osteoarticular’ OR ‘Bone Tuberculosis’ OR ‘Bone Tuberculoses’ OR ‘Tuberculoses, Bone’ OR ‘Tuberculosis, Bone’ OR ‘Joint Tuberculosis’ OR ‘Joint Tuberculoses’ OR ‘Tuberculoses, Joint’ OR ‘Tuberculosis, Joint’ OR ‘tuberculous arthritis’ OR ‘musculoskeletal tuberculosis’

#3 'tuberculous spondylitis'/exp

#4 ‘Spinal Tuberculoses’ OR ‘Spinal Tuberculosis’ OR ‘Tuberculoses, Spinal’ OR ‘Pott Disease’ OR ‘Disease, Pott’ OR ‘Pott's Disease’ OR ‘Disease, Pott's’ OR ‘Potts Disease’ OR ‘Pott's Paraplegia’ OR ‘tuberculous spondylitis’

#5 ‘Extrapulmonary tuberculosis’ OR ‘Extra pulmonary tuberculosis’

#6 #1 OR #2 OR #3 OR #4 OR #5

#7 Xpert OR genexpert

#8 #6 And #7

CNKI and Wanfang database:

#1 骨OR关节OR脊柱 OR肺外

#2 结核

#3 #1 AND #2

#4 Xpert OR genexpert

#5 #3 AND #4
