# Supplementary figures and images for: Diagnostic accuracy of the Xpert MTB/RIF assay for bone and joint tuberculosis: A meta-analysis
Source: PLoS One. 2019 Aug 22;14(8):e0221427. doi: 10.1371/journal.pone.0221427 (PMC6705841; doi:10.1371/journal.pone.0221427)

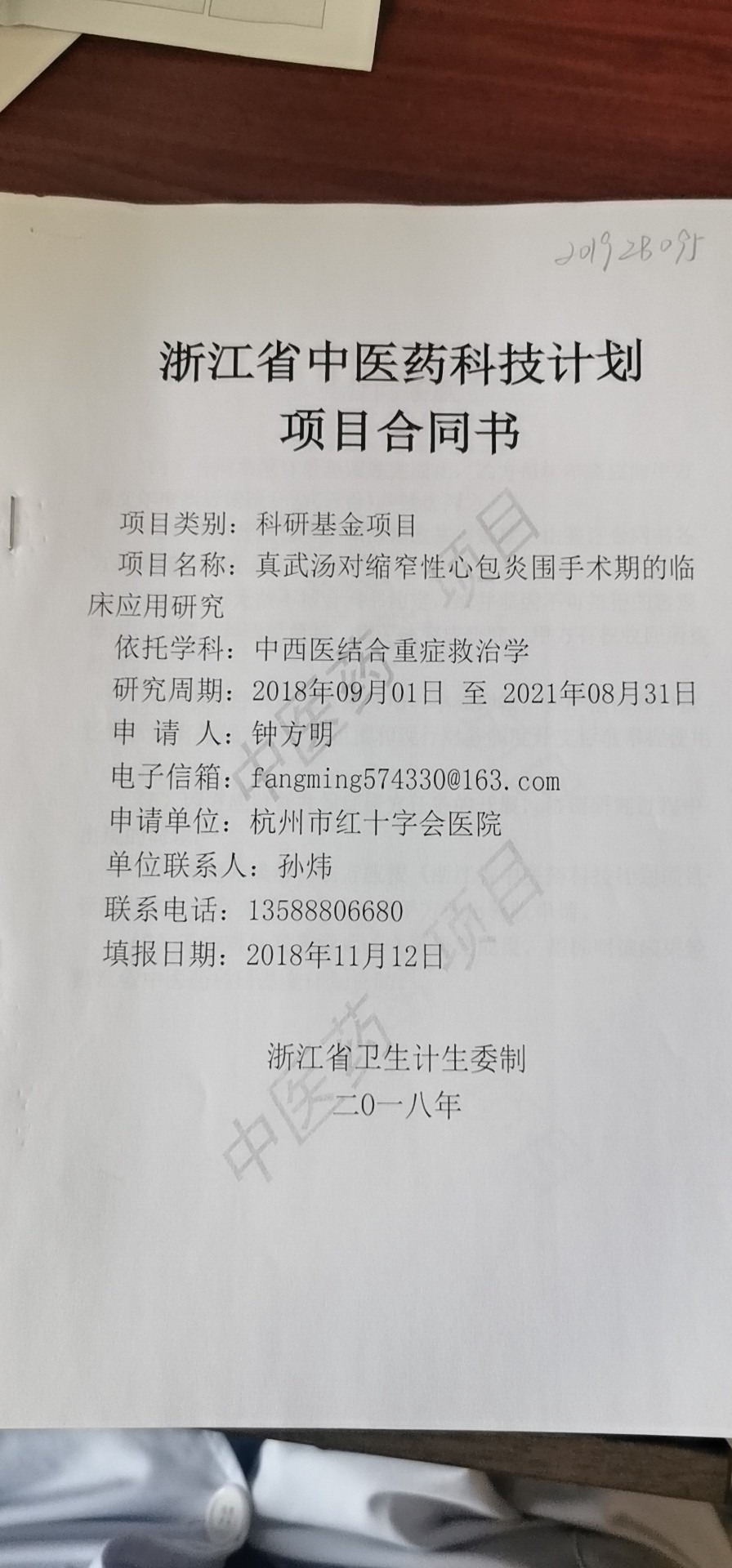

Supplement: S1 Supporting Information. Data — (ZIP) [file pone.0221427.s001.zip › S1 Supporting Information/funding/1.jpg]

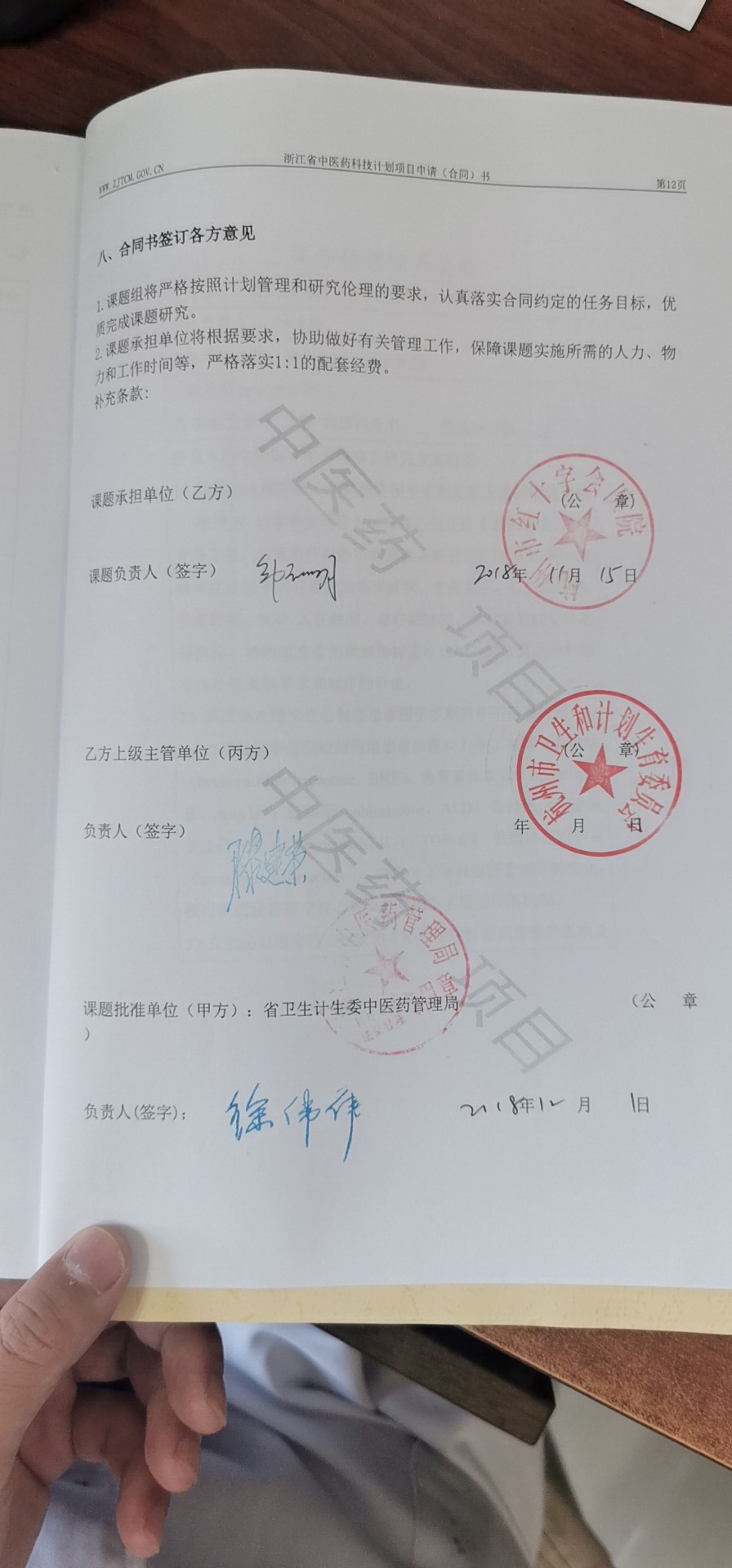

Supplement: S1 Supporting Information. Data — (ZIP) [file pone.0221427.s001.zip › S1 Supporting Information/funding/3.jpg]
