# Supplementary material for: Diagnostic accuracy of the Xpert MTB/RIF assay for bone and joint tuberculosis: A meta-analysis
Source: PLoS One. 2019 Aug 22;14(8):e0221427. doi: 10.1371/journal.pone.0221427 (PMC6705841; doi:10.1371/journal.pone.0221427)
Supplement: S1 Supporting Information. Data — (ZIP) [file pone.0221427.s001.zip › S1 Supporting Information/PRISMA 2009 flow diagram.doc]

**PRISMA 2009 Flow Diagram**

**Screening**

**Included**

**Eligibility**

**Identification**

Records identified through database searching
(n = 400)

Additional records identified through other sources
(n=0)

Records after duplicates removed
(n=128)

Records screened
(n=272)

Records excluded
(n =66)

Full-text articles assessed for eligibility
(n=206)

Full-text articles excluded, with reasons (n=180)

Review (n=61）

Not bone and joint tuberculosis (70)

Conference abstract (n=4)

No separate data(n=5)

Only sensitivity (n=5)

Not sensitivity (n=31)

Not English or Chinese (2)

Trial without result (2)

Records included in qualitative synthesis
(n=26)

Records included in quantitative synthesis (meta-analysis)
(n=26)
